# Variables influencing the prediction of fluid responsiveness: a systematic review and meta-analysis

**DOI:** 10.1186/s13054-023-04629-w

**Published:** 2023-09-20

**Authors:** Jorge Iván Alvarado Sánchez, Juan Daniel Caicedo Ruiz, Juan José Diaztagle Fernández, Luís Eduardo Cruz Martínez, Fredy Leonardo Carreño Hernández, Carlos Andrés Santacruz Herrera, Gustavo Adolfo Ospina-Tascón

**Affiliations:** 1 Fundación Santa Fe de Bogotá, Department of Intensive Care, Bogotá, Colombia; 2 Department of Physiology Sciences, Faculty of Medicine, Universidad Nacional de Colombia, Bogotá, Colombia; 3 Department of Internal Medicine, Fundación Universitaria de Ciencias de La Salud. Hospital de San José, Bogotá, Colombia; 4 Universidad de Los Andes, Bogotá, Colombia; 5 Department of Intensive Care, Fundación Valle del Lili, Cali, Colombia; 6 Translational Research Laboratory in Critical Care Medicine (TransLab-CCM), Universidad Icesi, Cali, Colombia

**Keywords:** Critical care, Fluid responsiveness, Pulse pressure variation, Stroke volume variation, Passive leg raising, End-expiratory occlusion test, Mini-fluid challenge

## Abstract

**Introduction:**

Prediction of fluid responsiveness in acutely ill patients might be influenced by a number of clinical and technical factors. We aim to identify variables potentially modifying the operative performance of fluid responsiveness predictors commonly used in clinical practice.

**Methods:**

A sensitive strategy was conducted in the Medline and Embase databases to search for prospective studies assessing the operative performance of pulse pressure variation, stroke volume variation, passive leg raising (PLR), end-expiratory occlusion test (EEOT), mini-fluid challenge, and tidal volume challenge to predict fluid responsiveness in critically ill and acutely ill surgical patients published between January 1999 and February 2023. Adjusted diagnostic odds ratios (DORs) were calculated by subgroup analyses (inverse variance method) and meta-regression (test of moderators). Variables potentially modifying the operative performance of such predictor tests were classified as technical and clinical.

**Results:**

A total of 149 studies were included in the analysis. The volume used during fluid loading, the method used to assess variations in macrovascular flow (cardiac output, stroke volume, aortic blood flow, volume‒time integral, etc.) in response to PLR/EEOT, and the apneic time selected during the EEOT were identified as technical variables modifying the operative performance of such fluid responsiveness predictor tests (*p* < 0.05 for all adjusted vs. unadjusted DORs). In addition, the operative performance of fluid responsiveness predictors was also influenced by clinical variables such as the positive end-expiratory pressure (in the case of EEOT) and the dose of norepinephrine used during the fluid responsiveness assessment for PLR and EEOT (for all adjusted vs. unadjusted DORs).

**Conclusion:**

Prediction of fluid responsiveness in critically and acutely ill patients is strongly influenced by a number of technical and clinical aspects. Such factors should be considered for individual intervention decisions.

**Supplementary Information:**

The online version contains supplementary material available at 10.1186/s13054-023-04629-w.

## Introduction

Fluid therapy is one of the first-line interventions used to reverse tissue hypoperfusion during acute circulatory failure. In general, fluid administration aims to increase cardiac output (CO), supposing that this will improve tissue perfusion and then cellular respiration and cell metabolism [1]. Nevertheless, excessive fluid administration may cause deleterious effects due to the induction of interstitial edema, impairment of microvascular flow, limitation of oxygen diffusion to the cells, increase in central venous pressure (which limits venous return), and increase in body distribution volumes. Indeed, fluid overload has been related to worse clinical outcomes, whereby fluids should be administered when a positive impact on macrohemodynamics is anticipated [2, 3].

A number of maneuvers have been described to assess fluid responsiveness in acutely and critically ill patients [2]. These include, among others, pulse pressure variation (PPV), stroke volume variation (SVV), variations in cardiac output or pulse pressure after passive leg raising (PLR), end-expiratory transitory apnea in mechanically ventilated patients (i.e., the end-expiratory occlusion test, EEOT), rapid infusion of a low volume of fluids (i.e., the mini-fluid challenge, MFC), and transitory increases in tidal volumes in patients with reduced pulmonary compliance subjected to protective mechanical ventilation (i.e., the tidal volume challenge, VTC) [4]. In general, such predictive tests of fluid responsiveness have shown acceptable operative performances in different clinical settings [5–18]. Nonetheless, heterogeneity of populations, variety of clinical scenarios, and technical differences to assess fluid responsiveness might complicate its application to specific individual clinical contexts. [14, 15, 18]. Only a few studies have included subgroup analyses that allow the assessment of sources of heterogeneity or the evaluation of variations in the operative performance of individual tests [5, 6, 15–18].

The performance of predictor tests to assess fluid responsiveness might be affected by a number of clinical and technical factors [17], which could lead to false positive or negative results with the subsequent increased risk of fluid overload or hypovolemia when such predictors are used to guide individual decisions. Although it is known that certain physiological variables might affect the performance of predictors of fluid responsiveness, few meta-analyses or meta-regressions have evaluated the variations in performance to predict fluid responsiveness in particular subgroups [5, 6, 15–18]. Therefore, we aim to systematically assess the impact of clinical and technical variables potentially modifying the performance of predictor tests of fluid responsiveness in critically ill and perioperative adult patients using subgroup analysis and meta-regression.

## Methods

### Protocol

This meta-analysis was conducted following the Preferred Reporting Items for Systematic Reviews and Meta-Analysis (PRISMA) for diagnostic test accuracy (DTA) guidelines [19]. The complete predefined protocol was registered in PROSPERO (registration number CRD42021266950) (https://www.crd.york.ac.uk/prospero/display_record.php?ID=CRD42021266950) in August 2021.

### Search strategy and data extraction

A comprehensive search strategy was conducted using the Medline and Embase databases between January 1999 and February 2023. Data extraction and eligibility assessment were standardized and independently performed by two reviewers (J.I.A.S. and J.D.C.R.). Reference lists of selected manuscripts were also manually reviewed to search for potential studies not retrieved by the original search. No language restriction was applied. The complete search strategy and the terms used are available in the protocol uploaded to PROSPERO (registration number CRD42021266950).

### Study selection and inclusion criteria

Studies were selected according to the PICOS statement as follows:

- *P*-Patients and setting: We selected studies including critically ill and acutely ill perioperative patients. We excluded studies conducted in the emergency room and those including patients < 18 years of age and pregnant women.
- *I*-index test: We selected studies evaluating the operative performance of PPV, SVV, PLR, EEOT, MFC, and VTC as predictors of fluid responsiveness. Studies evaluating PPV and SVV performance were selected when they included patients under mechanical ventilation without respiratory effort and arrhythmias. Meanwhile, studies evaluating PLR-induced changes in both cardiac output and pulse pressure, including patients with intra-abdominal hypertension (explicit definition), were not selected.
- C-comparison or reference standard: Only studies including an explicit definition of fluid responsiveness (reference standard) after fluid loading or another maneuver (such as PLR or EEOT) were incorporated into the analysis.
- O-outcomes or target condition: We selected only studies reporting data about the operative performance of any fluid responsiveness test. Some studies had several data points on operative performance; in such cases, all data regarding operative performance were included.
- S-studies: We selected prospective studies. Meanwhile, case reports, studies with incomplete data, studies conducted in animals, studies assessing patients with heart failure, those including patients under one-lung ventilation or open chest surgery, and retrospective studies were excluded from the analysis.

### Study selection and data collection process

Two authors (J.I.A.S. and J.D.C.R.) independently reviewed the titles and potentially eligible abstracts. Studies fulfilling the inclusion criteria were pooled in individual extraction sheets to be ultimately compared. Discrepancies about inclusion, quality, adequacy of data, and final classification of eligible studies were resolved by consensus among the authors.

### Data Items

Data extracted from eligible studies included year of publication, authors, number of participants, sensitivity, specificity, area under the curve (AUC); method employed to assess preload dependency (i.e., excitation method): fluid challenge, PLR, EEOT and Trendelenburg; technique used to assess the selected excitation method; variable employed for appraising fluid responsiveness (i.e., the peri-excitation macrohemodynamic variable), and clinical setting. Variables potentially modifying the operative performance of predictors of fluid responsiveness were classified as technical and clinical. Technical variables included fluid loading > or ≤ 4 mL/kg; hemodynamic variables were used to determine the PLR/EEOT response, which were separated into two groups: (a) CO direct measurements: aortic blood flow (ABF), CO/cardiac index (CI), stroke volume (SV)/stroke volume index (SVI), and velocity–time integral (VTI); and (b) CO surrogates: pulse pressure (PP), SVV and PPV. Other technical variables included the time lapse of expiration during the EEOT maneuver (≤ 15 s, > 15 – < 30 s and ≥ 30 s); the threshold used to define fluid responsiveness (< or ≥ 10%); the use or not of thermodilution as a gold standard to measure CO; and whether the volume of MFC was considered a part of the fluid load during the FR assessment. Meanwhile, clinical variables included the type of patient, driving pressure, level of positive end-expiratory pressure (PEEP), tidal volume (Vt), total respiratory compliance, supine vs. prone position ventilation, and norepinephrine dose (< or ≥ 0.3 mcg/kg/min).

### Risk of bias in individual studies

The quality of eligible studies was assessed according to the Quality Assessment of Diagnostic Accuracy Studies 2 (QUADAS-2) using 4 domains: patient selection, index test, reference standard, and flow and time. Each area was evaluated for risk of bias and classified as low, high, or unclear. Moreover, the risk of applicability was considered in three domains: patient selection, index test, and reference standard, which were in turn assessed as low, high, or unclear [20].

### Statistical analysis

#### Analysis of individual studies

We computed the sensitivity, specificity, and diagnostic odds ratio (DOR) using a contingency table. In certain studies, operative performance was assessed in multiple subgroups, including different ventilation parameters, techniques used to assess fluid responsiveness, cutoff values to consider the test as positive, and methods for evaluating fluid response. In such cases, all the available data related to the operative performance were incorporated.

DORs were calculated as described elsewhere (21). Higher DORs identify predictors with higher sensitivity and specificity combined with lower rates of false positives and negatives.

#### Analysis of summary measures

Fitted data of sensitivity, specificity, and AUC were assessed by using bivariate and hierarchical analyses. The summary receiver operating characteristic (ROC) curve was assessed by the Rutter and Gatsonis method, while the AUC was graduated according to Fisher et al. [21]. Unadjusted DOR was calculated by using a random-effects model. Heterogeneity was evaluated by Cochran Q statistics; its effect was quantified by using inconsistency (*I*^2^).

#### Analysis of risk of bias across studies

Asymmetry was assessed by Egger tests [22]. Publication bias was fitted by the trim-and-fill method [23]

#### Subgroup analysis and meta-regression

Adjusted DORs were calculated by subgroup analyses (inverse variance method) and meta-regression (test of moderators) for all technical and clinical variables potentially influencing the operative performance of predictors of fluid responsiveness, as described above. Meta-regression was especially used to assess continuous variables such as Vt, lung compliance, PEEP, and driving pressure.

### Additional analysis

A meta-regression was performed based on the quality assessment of all studies included (according to the QUADAS-2).

The sensitivity analysis is summarized in Table 4. It encompasses the measurement methods employed to assess the excitation method: continuous thermodilution/pulmonary artery catheter, transpulmonary thermodilution, calibrated/non-calibrated pulse contour analysis, bioimpedance/bioreactivity, and esophageal Doppler. Additionally, clinical settings, including cardiovascular, neurological, neurosurgical, other surgical, and sepsis contexts, were also considered. Furthermore, hemodynamic variables used to determinate the response to passive leg raising (PLR) or the end-expiratory occlusion test (EEOT) encompassed cardiac output, aortic blood flow, stroke volume, and velocity–time integral. Such variables were included as reported in each study and were identified as the primary sources of heterogeneity.

The Grading of Recommendations Assessment, Development, and Evaluations (GRADE) was performed as a diagnostic test [24]. The threshold effect was evaluated by Spearman´s rank correlation coefficient and by the Moses-Shapiro-Littenberg method [25]. Data were analyzed using R version 3.4.3 with the Meta-Analysis of Diagnostic Accuracy (MADA) and meta-packages. The data are expressed as a value (95% confidence interval [CI]). *P* < 0.05 was considered statistically significant.

## Results

### Study selection

**Fig. 1:**
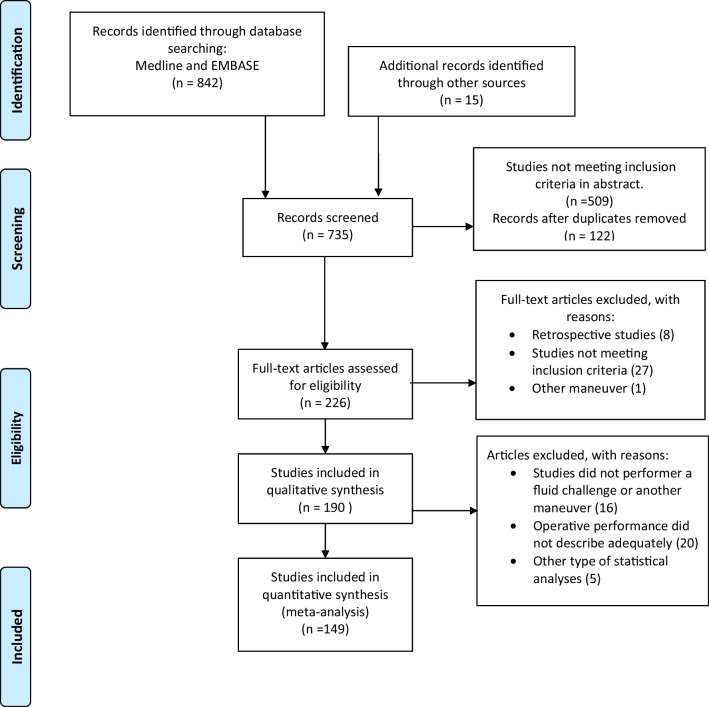
Study selection

From 857 preliminary records identified from the EMBASE and Medline databases and their reference lists and after removing duplicated records and studies not fulfilling the inclusion criteria, a total of 149 studies were finally included in the analysis (Fig. 1).

### Study characteristics, risk of bias, and syntheses of results

A total of 6488 patients were included in the meta-analysis, with 7350 fluid challenges performed and an average fluid responsiveness of 53%. General characteristics of the included studies are described in the Additional file 1: Appendix: Table S1, while operative performance for different fluid responsiveness predictors is depicted in the Additional file 1: Appendix (Tables S2 – S7).

**Fig. 2:**
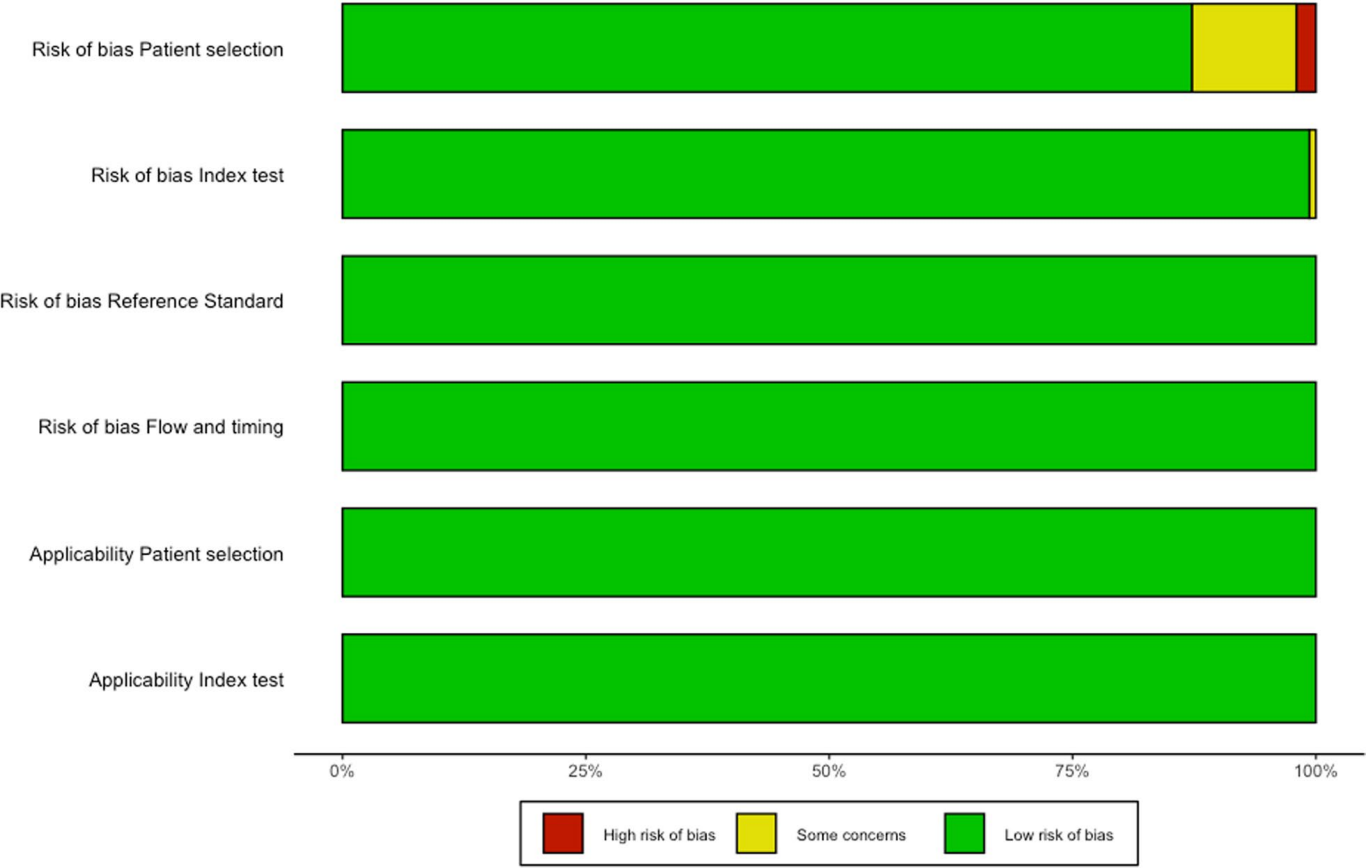
Risk of bias

The risk of bias is depicted in Fig. 2 and the Additional file 1: Appendix (Table S8), while the operative performance of fluid responsiveness predictors is depicted in Table 1. The asymmetry assessment is depicted in the Additional file 1: Appendix (Table S9), while the asymmetry fitted by the trim-and-fill method is depicted in the Additional file 1: Appendix (Table S10). Studies assessing PPV, SVV, PLR, EEOT, and VTC showed significant asymmetry (*p* < 0.05).

**Table 1:** Unadjusted operative performance of predictor of fluid responsiveness evaluated

Predictor of fluid responsiveness	AUC	Sensitivity (95% CI)	Specificity (95% CI)	Unadjusted DOR (95% CI)	*I*^2^ (95% CI) (%)	*Q*	*p* value
PVV	0.84	0,77(0.74–0.80)	0.79(0.76–0.82)	16.6 (12.9 – 21.7)	52.8	216.2	< 0.01
SVV	0.83	0.77(0.73–0.80)	0.78(0.74–0.82)	14.4(10.7–19.4)	52.9	144.42	< 0.01
PLR	0.84	0.77(0.73–0.80)	0.80(0.75–0.83)	22.6(17.0–30.2)	53.7	181.85	< 0.01
EEOT	0.83	0.76(0.70–0.81)	0.77(0.71–0.83)	17.8(10.7–30.0)	52.6	107.9	< 0.01
MFC	0.90	0.85(0.79–0.90)	0.84(0.78–0.88)	37.3(21.8–64.0)	30.0	21.4	0.12
VTC	0.89	0.86(0.81–0.91)	0.80(0.74–0.85)	46.6(26.7–81.3)	28.5	33.25	0.09

*AUC* The area under curve, *I*^2^, Inconsistency, *PPV* Pulse pressure variation, *Q* Cochran Q statistics, *SVV* Stroke volume variation, *PLR* Passive leg raising, *EEOT* End-expiratory occlusion test, *MFC* Mini-fluid challenge, *VTC* Tidal volume challenge

Values are expressed as pooled data (95% confidence interval)

*P*–value was obtained by heterogeneity test

In general, heterogeneity was moderate for PPV, SVV, PLR, and EEOT (*p* = 0.01), and the studies that evaluated MFC and VTC were homogeneous (*p* > 0.05).

### Subgroup analysis and meta-regression

Subgroup analysis and meta-regression are shown in Tables 2 and 3 and the Additional file 1: Appendix (Table S11).

**Table 2:** Adjusted operative performance of the technical variables for predictors of fluid responsiveness evaluated

Subgroup	Predictor evaluated	Number of studies	Unadjusted DOR (95%CI)	Adjusted DOR (95% CI)	*P* value by subgroup analysis	*P* value by meta-regression	*I*^2^(%)
*Fluid loading* > *4 ml/kg*							
Yes No	PPV	85 14	16.6 (12.9 – 21.7)	19.4(14.5–26.0) 8.1(4.3–15,2)	< 0.01	0.01 < 0.01	49.8
*Fluid loading* > *4 ml/kg*							
Yes No	SVV	45 21	14.4(10.7–19.4)	12.4(9.6–16.1) 11.7(8.6–15.7)	0.63	NA	54.0
*Fluid loading* > *4 ml/kg*							
Yes No	PLR	55 16	22.6(17.0–30.2)	26.0(18.1–37.3) 13.0(7.3–23.2)	0.045	0.05 < 0.01	53.0
*Fluid loading* > *4 ml/kg*							
Yes No	EEOT	23 10	17.8(10.7–30.0)	24.4(13.3–44.8) 6.81(3.0–15.2)	< 0.01	< 0.01 < 0.01	57
Fluid loading > 4 ml/kg							
Yes No	MFC	14 2	37.3(21.8–64.0)	35.7(22.0–58.1) 30.2(11.0–83.5)	0.09	NA	35.3
Fluid loading > 4 ml/kg							
Yes No	VTC	13 4	46.6(26.7–81.3)	30.1(17.2–52.5) 127.2(36.3–446.16)	0.06	NA	10.3
*Hemodynamic variable to determine PLR response*							
Direct measurements (ABF, CO, CI, SV, SVI, VTI) Surrogate	PLR	47 36	22.6(17.0–30.2)	32.3(21.7–48.1) 15.3(10.2–23.0)	< 0.01	< 0.01 < 0.01	53.7
*Hemodynamic variable to determine EEOT response*							
Direct measurements (ABF, CO, CI, SV, SVI, VTI) Surrogate	EEOT	23 13	17.8(10.7–30.0)	36.7(19.7–68.2) 6.8(3.4–13.7)	< 0.01	< 0.01 < 0.01	57.2
*Lapse of time selected for EEOT*							
≤ 15 s > 15 – < 30 s ≥ 30 s	EEOT	22 11 3	17.8(10.7–30.0)	36.8(18.9–72.0) 10.1(4.6–22.0) 4.5(1.0–18.9)	< 0.01	0.01 0.23 < 0.01	59.3

*DOR* Diagnostic odd ratio, *I*^2^ Inconsistency. *NA* Not applicable, *PPV* Pulse pressure variation, *PLR* Passive leg raising, *EEOT* End-expiratory occlusion test, *MFC* Mini-fluid challenge, *VTC* Tidal volume challenge, *ABF* Aortic blood flow, *CO* Cardiac output, *CI* Cardiac index, *SVI* Stroke volume index, *SV* Stroke volume, *VTI* Velocity–time integral. Values are expressed as pooled data (95% confidence interval)

**Table 3:** Adjusted operative performance of the clinical variables for predictors of fluid responsiveness evaluated

Subgroup	Predictor evaluated	Number of studies	Unadjusted DOR (95%CI)	Adjusted DOR (95% CI)	*P* value by subgroup analysis	*P* value by meta-regression	*I*^2^(%)
PEEP	EEOT	38	17.8(10.7–30.0)	DOR = 1.06 + 1.21 (cmH2O PEEP)	NA	< 0.01	53.4
Norepinephrine dose ≥ 0.3 mcg/kg/min							
Yes No	PPV	25 10	16.6 (12.9 – 21.7)	14.1(8.5–23.4) 18.0(7.8–41.3)	0.63	NA	50.9
Norepinephrine dose ≥ 0.3 mcg/kg/min							
Yes No	SVV	8 5	14.4(10.7–19.4)	13.3(4.8–36.6) 29.4(7.8–111.2)	0.34	NA	60.6
Norepinephrine dose ≥ 0.3 mcg/kg/min							
Yes No	PLR	24 11	22.6(17.0–30.2)	49.9(27.6–90.1) 16.2(7.7–34.2)	0.02	0.02 < 0.01	61.2
Norepinephrine dose ≥ 0.3 mcg/kg/min							
Yes No	EEOT	19 3	17.8(10.7–30.0)	58.7(28.6–120.4) 11.5(3.0–44.2)	0.03	0.04 < 0.01	45.2

*DOR* Diagnostic odd ratio, *I*^2^ Inconsistency. *PPV* Pulse pressure variation, *PEEP* Positive end-expiratory pressure, *SVV* Stroke volume variations, *EEOT* End-expiratory occlusion test; *PLR* Passive leg raising. Values are expressed as pooled data (95% confidence interval)

### Technical variables

The volume of fluid administered had a significant impact on the predictive performance of several hemodynamic variables, namely pulse pressure variation (PPV), passive leg raising (PLR), and the end-expiratory occlusion test (EEOT). When fluid loading exceeded 4 ml/kg, the predictive performance of such fluid responsiveness predictors increased, as evidenced by the increase in the diagnostic odds ratio (DOR). Conversely, when fluid doses were ≤ 4 ml/kg, the opposite trend was observed, resulting in a decrease in the DOR values for all three predictors (*p* < 0.05) (Table 2).

The hemodynamic variables used to determine the PLR/EEOT response were found to significantly influence their predictive performance. Direct macro-flow measurements such as aortic blood flow (ABF), cardiac output (CO) or cardiac index (CI), stroke volume (SV) or stroke volume index (SVI), and velocity–time integral (VTI) increased its predictive performance (Table 2). Conversely, using surrogates of CO led to a decrease in predictive performance for both PLR and EEOT. Moreover, the EEOT predictive performance was found to increase when the time lapse for the EEOT maneuver was ≤ 15 s (*p* < 0.01), while its predictive performance significantly decreased when the time lapse exceeded 30 s (*p* < 0.01) (Table 2).

The operative performance related to other variables evaluated is shown in the Additional file 1: Appendix (Table S11).

### Clinical variables

As expected, progressively higher positive end-expiratory pressure (PEEP) values improved the ability of the end-expiratory occlusion test (EEOT) to predict fluid responsiveness. In fact, increasing PEEP by just 1.21 cmH2O led to a significant improvement in EEOT performance (*p* < 0.01) (Table 3).

Higher doses of norepinephrine (≥ 0.3 mcg/kg/min) were related to significant improvements in the performance of PLR and EEOT to predict fluid responsiveness, while lower doses had the opposite effect (Table 3). Nevertheless, norepinephrine dose was not related to significant changes in the performance of PPV and SVV (*p* > 0.05) (Table 3).

Additional analyses of the sources of variability are summarized in the Additional file 1: (Table S11).

### Additional analysis

There was no threshold effect for any predictor (*p* > 0.05), as evaluated by both the rho correlation coefficient and the Moses–Shapiro–Littenberg test. The accuracy of PPV as a diagnostic measure was deemed moderately reliable because the results from the studies included lack precision and might not be applicable to all clinical settings. Conversely, the accuracy of other predictors was deemed highly reliable. Additional information on the GRADE assessment can be found in Appendix Table S12-Table S17 of the Additional file 1.

### Sensitivity analysis

Sensitivity analysis did not retrieve significant changes in the operative performance of any of the fluid responsiveness predictors evaluated according to the methodological quality (QUADAS-2) (*p* > 0.05) (Table S18 of the Additional file 1). However, sensitivity analysis (Table 4) revealed several sources of heterogeneity (Table 4). Such sources of heterogeneity accounted for varying percentages of heterogeneity in each predictor. More details on the sensitivity analysis can be found in Additional file 1: Appendix Table S19.

**Table 4:** Sensitivity analysis of predictor of fluid responsiveness based on ﻿the reference standard used, type of patient, hemodynamic variable to determine PLR/EEOT response, device ued to measure PLR response and the number of patients included in each study

Risk of bias within studies	Predictor	Number of studies	Adjusted diagnostic odds ratios (95% CI)	P value by test for subgroup	*I*^2^ (%)	*R*^2^ (%)
Measurement method to assess excitation method	SVV					
PAC Esophageal Doppler TTE TEE Impedance PCA (LiDCO) NC-PCA(ModelFlow) NC-PCA(MostCare) NC-PCA(PiCCO) TPTD(PiCCO) PAC(Vigilance) NC-PCA(Vigileo)		4 8 1 3 6 1 2 1 5 16 2 20	17.9(6.7–47.9) 6.0(3.2–11.4) 4.6(1.1–19.5) 4.6(1.5–13.9) 79.9(34.1–187.9) 697.0(22.8–21,293.6) 34.6(4.3–284.1) 5.06(1.3–20.5) 14.0(6.5–30.1) 9.2(5.6–15.2) 8.9(2.2–36.4) 20.0(12.3–32.5)	0.02	29.1	61.8
Device used to measure PLR response	PLR					
C-TD Capnography Doppler Esophageal Doppler TTE TTE/PCA (PiCCO) Bioimpedance technique Others C-PCA(PiCCO) Arterial pressure Photoplethysmography technology Abdominal ultrasonography NC-PCA(Vigileo)		1 2 5 2 24 2 2 3 22 12 1 4 3	10.1(1.8–55.7) 118.9(15.9–887.2) 50.4(20.7–122.7) 169.7(25.9–1111.88) 22.7(14.8–34.7) 66.8(28.9–154.7) 83.4(10.7–652.7) 21.3(6.3–72.4) 17.8(10.8–29.2) 6.4(4.10–10.0) 38.7(7.2–207.2) 16.1(4.8–54.3) 73.1(16.4–325.6)	< 0.01	21.4	74.7
Measurement method to assess excitation method	PLR					
Esophageal Doppler TTE TTE/TPTD(PiCCO) Bioimpedance technique C-PCA(PiCCO) TPTD(PiCCO) Ultrasonic cardiac output monitor PAC (Vigilance) NC-PCA(Vigileo)		6 31 2 2 12 21 2 1 6	30.2(11.2–81.1) 24.4(15.9–37.5) 67.0(21.7–206.3) 88.1(10.5–742.46) 28.3(13.1–61.2) 13.6(8.0–23.1) 63.0(13.1–301.4) 10.1(1.4–75.5) 8.5(4.0–18.2)	0.02	38.9	38.9
Measurement method to assess excitation method	EEOT					
Esophageal Doppler TEE TTE NC-PCA(MostCare) C-PCA(PiCCO) TPTD(PiCCO) NC-PCA(Pulsioflex/ProAQT) NC-PCA(Vigileo)		2 3 2 4 1 16 4 4	8.3(1.8–37.7) 11.2(3.5–36.3) 19.0(3.7–96.4) 15.0(5.2–43.2) 96.2(11.1–831.4) 64.9(30.0–140.3) 6.2(2.1–18.3) 3.1(1.1–8.7)	0.01	43.2	59.3
Clinical setting	EEOT					
Cardiovascular Neurologic Neurosurgical Surgical Septic		3 2 4 10 17	11.1(3.6–34.8) 18.5(3.8–90.3) 20.5(6.2–68.2) 5.0(2.71–9.2) 67.2(32.9–137.4)	0.01	42.1	63.5
Hemodynamic variable to determine EEOT response ETCO2 FTC CI SVI Others PI PP Cardiac Peak velocity PPV SV VTI	EEOT	1 1 13 3 1 2 3 3 2 4 3	6.1(1.4–25.7) 15.0(2.3–99.6) 105.5(50.6–219.2) 9.1(4.6–18.4) 8.4(1.4–48.8) 4.7(1.8–12.4) 4.9(2.3–10.4) 8.1(3.3–19.4) 4.7(2.0–10.6) 4.2(2.0–10.6) 83.9(26.6–264.3)	0.01	11.4	92.9

*I*^2^ Inconsistency, *R*^2^ Amounts of heterogeneity, C-TD, continuous thermodilution, C-PCA, calibrated pulse contour analysis, *CO* Cardiac output, *CI* Cardiac index, *EEOT* End-expiratory occlusion test, *NC-PCA* Non-calibrated pulse contour analysis, *PAC* Pulmonary artery catheter, *PLR* Passive leg raising, *PPV* Pulse pressure variation, *SV* Stroke volume; *SVI* Stroke volume index, *SVV* Stroke volume variation, *TEE* Transesophageal echocardiography, *TPTD* Transpulmonary thermodilution, *TTE* Transthoracic echocardiography, *VTI* Velocity time integral

## Discussion

This systematic review and meta-analysis highlights the impact of some technical and clinical variables on the operative performance of predictors of fluid responsiveness commonly used in clinical practice. A fluid loading > 4 ml/kg, the type of hemodynamic variable used to assess the response to PLR/EEOT, the apneic time during the EEOT maneuver, the PEEP level used during EEOT, and the doses of norepinephrine used at the FR assessment were identified as the main technical/clinical variables influencing the operative performance of commonly used fluid responsiveness predictors. Consequently, such variables should be carefully considered when making individual decisions.

Previous meta-analyses have assessed the value of fluid responsiveness predictors in various clinical scenarios, demonstrating their overall good performance [5–18]. However, heterogeneity of the studies included in such meta-analyses hinders their applicability to clinical practice. To address this issue, subgroup analysis and meta-regression are recommended to determine sources of heterogeneity and assess changes in operative performance within each subgroup [26]. Several meta-analyses conducted subgroup analyses and recognized the impact of some clinical variables on operative performance to predict fluid responsiveness [5, 6, 15–18]. Unlike some authors using bivariate analysis to assess operative performance [16, 17], we evaluated DORs as a summary measure, and we employed meta-regression to examine quantitative variables.

The operative performance of PPV, PLR, and EEOT was significantly improved in cases in which fluid loading was > 4 mL/kg, which agrees with previous observations showing that increases in fluid loading from 1 to 4 mL/kg enhanced the rate of fluid responders from 20 to 65% [27]. Nevertheless, others have not found significant variations in the performance of EEOT caused by different volumes of fluid loading [18], while its influence on SVV and PLR performance is difficult to evaluate because of the high heterogeneity of the studies included [17].

Direct estimates of macrovascular blood flow, such as CO/CI, ABF, SV/SVI, and VTI, are related to significantly better operative performance of PLR/EEOT. In contrast, using CO surrogates such as pulse pressure (PP), SVV, and PPV led to a decrease in operative performance. This phenomenon may be due to the relationship between CO surrogates and other physiological variables [28], such as PP, which is linked to arterial compliance, impedance, and wave reflection [29]. These findings align with those of previously published studies [30].

The duration of the respiratory hold constitutes a critical technical variable that demands careful consideration during the execution of this test. Notably, the operative performance displays an augmentation when the EEOT duration is ≤ 15 s, while a decline is observed when it extends to ≥ 30 s. This trend might be attributed to the fact that the maximal alteration in venous return and cardiac output typically materializes within the initial 15 s, with subsequent reductions occurring due to the finite nature of this change [31]. Moreover, it is imperative to acknowledge that a persistent reduction in pleural pressure has the potential to elevate left ventricular afterload [32], especially in individuals with myocardial dysfunction [33]. This phenomenon, in turn, could lead to a decrease in stroke volume when the EEOT maneuver surpasses the 30-s mark. While this finding aligns with physiological plausibility, it is worth noting that, to the best of our knowledge, no clinical trials or other meta-analyses have reported a substantial alteration in the operative performance of EEOT based on its duration [15, 18]. We attribute this variance to three key factors. First, our evaluation encompassed three distinct subgroups, whereas the referenced meta-analyses examined two subgroups. Second, two clinical studies that employed a respiratory hold duration of ≥ 30 s were excluded from the mentioned meta-analyses [34, 35]. Last, it is noteworthy that these studies reported a notably low operative performance for EEOT [34, 35].

Although some authors have suggested that including the volume of the MFC within the fluid loading might change its operative performance [36], our meta-analysis did not confirm such data.

Our findings reported a positive relationship between EEOT operative performance and PEEP, which could be explained for two reasons. First, increasing PEEP could improve pulmonary compliance (by alveolar recruitment) due to an increase in cyclic changes in intrathoracic pressure induced by EEOT, thus increasing its operative performance [37]. Second, the use of a high PEEP increases right ventricular afterload and right atrial pressure, which would lead to a decrease in venous return, finally increasing preload dependence and increasing its operative performance. Our findings did not agree with the results reported by Silva et al. [37], who did not find a relationship between EEOT operative performance and PEEP. This difference could be explained by two reasons. First, they only evaluated patients with acute respiratory distress syndrome (ARDS), while we also included studies that evaluated patients without ARDS. Second, they did not find that increasing PEEP changed the driving pressure (plateau pressure – PEEP); therefore, the changes in intrathoracic pressure induced by such an increase were not achieved, and the preload dependence did not change. Silva et al. concluded that EEOT operative performance remains reliable when PEEP is increased in ARDS patients, but the EEOT performance could change in patients in whom driving pressure increases; however, we did not find that driving pressure changes the EEOT operative performance (see Supplement Material Appendix; Table S11); therefore, the PEEP itself is a determinant of the hemodynamic effects of EEOT. Other meta-analyses have not found this finding [15, 18, 38], which could be due to our study assessing PEEP as a continuous variable (by meta-regression), while other meta-analyses evaluated PEEP as a categorical variable (by bivariate analysis). Finally, we did not find that Vt changes the EEOT operative performance (see Additional file 1: Appendix; Table S11).

Our data also suggest that doses of norepinephrine > 0.3 mcg/kg/min result in significant increases in the operative performance of PLR and EEOT. Previous studies have described that using norepinephrine is associated with fluid responsiveness [39]. The likelihood of exhibiting a positive fluid response is intricately linked to factors such as the mean systemic filling pressure (MSFP) and the interplay between stressed and unstressed volumes. In the context of vasoplegic states, the unstressed volume tends to rise, while both the stressed volume and MSFP decline. Consequently, there is a decrease in preload and stroke volume. Interestingly, these states may not manifest a fluid response since the introduction of fluid merely results in an elevated unstressed volume, lacking a corresponding rise in MSFP. As a result, the false negative rate of certain predictors may escalate, subsequently diminishing their operative performance during vasoplegic conditions [40]. The introduction of norepinephrine contributes to an increase in stressed volume and MSFP. This leads to a boost in venous return and stroke volume following a fluid load [41]. Remarkably, the relationship between operative performance and norepinephrine use has not been previously documented, as per our knowledge.

Our meta-analysis has several limitations. First, while the diagnostic odds ratio (DOR) summarizes the relationship between sensitivity and specificity, it does not indicate the direction of the relationship (which may vary among different predictors), even at similar DOR values [42]. Second, some special clinical conditions were not evaluated (i.e., patients with intra-abdominal hypertension), which limits the generalizability of our results. Third, a majority of predictors exhibited a moderate level of heterogeneity. However, our analysis pinpointed various sources of this heterogeneity, including disparities in the measurement methods used to evaluate the excitation method. Fourth, it is noteworthy that we did not explore other factors that could influence fluid responsiveness, such as the impact of medications such as analgosedation. These medications can potentially affect the autonomic nervous system, thereby potentially modifying the sympathetic response to fluid loading. It is important to note that the use of such medications was infrequently reported in most of the included studies, adding complexity to their potential impact on the observed outcomes. Finally, we did not investigate the connection between fluid responsiveness and tissue perfusion, a clinical aspect that we consider of utmost importance.

## Conclusions

Based on our findings, we recommend conducting a PLR/EEOT test with direct CO measurement. For the EEOT, the respiratory hold should be limited to less than 15 s. Physicians should be aware that higher positive end-expiratory pressure (PEEP) levels enhance the performance of EEOT. Additionally, the use of norepinephrine has been observed to enhance the operative performance of external fluid loading maneuvers, such as PLR and EEOT. Finally, when performing a fluid challenge, a volume of less than 4 ml/kg is suggested.

### Supplementary Information

**Additional file 1.** Supplement Material Appendix.

## Data Availability

The datasets used and/or analyzed during the current study are available from the corresponding author upon reasonable request.

## Abbreviations

ABF: Aortic blood flow
APACHE II: Acute physiology and chronic health evaluation score II
AUC: Area under the curve
CI: Cardiac index
CO: Cardiac output
cIVC: Inferior vena cava collapsibility index
DVpeakP: Brachial artery peak velocity
Delta-PI: Delta perfusion index
delta-VF: Peak velocity of femoral artery flow
DORs: Diagnostic odds ratios
EEOT: End-expiratory occlusion test
ETCO2: End-tidal carbon dioxide
FR: Fluid responsiveness
FPR: Predictor of fluid responsiveness
FTC: Flow time corrected
IVC max: Maximum inferior vena cava diameter
OP: Operative performance
PEEP: Positive end-expiratory pressure
PI: Perfusion index
PP: Pulse pressure
PRISMA: Preferred Reporting Items for Systematic Reviews and Meta-Analysis
PLR: Passive leg raising
PP: Pulse pressure
PPV: Pulse pressure variation
PVI: Respiratory variation of PI
SAPS II: Simplified acute physiology score II
SV: Stroke volume
SVI: Stroke volume index
SVV: Stroke volume variation
VTI: Velocity–time integral
Vpeak: Peak velocity of the aortic velocity signal
