# Supplementary material for: Variables influencing the prediction of fluid responsiveness: a systematic review and meta-analysis
Source: Crit Care. 2023 Sep 20;27:361. doi: 10.1186/s13054-023-04629-w (PMC10510289; doi:10.1186/s13054-023-04629-w)
Supplement: Supplementary file 1 — Additional file 1. Supplement Material Appendix. [file 13054_2023_4629_MOESM1_ESM.pdf]

Variables influencing prediction of fluid responsiveness: a systematic review and metanalysis.

Supplement Material Appendix

Jorge Iván Alvarado Sánchez[1, 2] **(ORCID: 0000-0003-4320-3150)**

Juan Daniel Caicedo Ruiz[2]

Juan José Diaztagle Fernández[2,3]

Luís Eduardo Cruz Martínez[2]

Fredy Leonardo Carreño[4]

Carlos Andrés Santacruz Herrera[1]

Gustavo Adolfo Ospina-Tascón [5,] **6 (ORCID: 0000-0001-9370-3298)**

1. Department of Intensive Care, Fundación Santa Fe de Bogotá, Bogotá Colombia.
2. Department of Physiology Sciences, Faculty of Medicine, Universidad Nacional de Colombia, Bogotá, Colombia.
3. Fundación Universitaria de Ciencias de la Salud, Bogotá, Colombia. Department of Internal Medicine, Hospital de San José, Bogotá Colombia.
4. Universidad de Los Andes, Bogotá, Colombia.
5. Department of Intensive Care, Fundación Valle del Lili, Cali, Colombia.
6. Translational Research Laboratory in Critical Care Medicine (TransLab-CCM), Universidad Icesi, Cali, Colombia

Corresponding author: Jorge Iván Alvarado Sánchez: Jialvarados@unal.edu.co

Study	Year	Sample Size	Number of Fluid Challenges	Setting	Excitation method	Measurement method to assess excitation method	Peri-excitation macrohemodynamic variable	Method Used to Measure Predictor	Predictor Of Fluid Responsiveness evaluated	Type of fluid used	Definition of Fluid Responsiveness	Fluid Responsiveness Rate
Michard et al (1)	2000	40	40	SEPSIS	FC (500 ml)	PAC	CI	Arterial tracing measurement*	PPV	Colloid	15%	0.4
Berkenstadt et al (2)	2001	15	140	NEUROSURGICAL	FC (100 ml)	C-PCA (PiCCO)	SV	PCA (PiCCO)	SVV	Colloid	5%	0.5
Kramer et al (3)	2004	21	21	CARDIOVASCULAR	FC (500 ml)	PAC	CO	Arterial tracing measurement*	PPV	PRBC	12%	0.28
De Backer et al (4)	2005	27	27	SEPSIS	FC (500-1000 ml)	PAC	CI	Arterial tracing measurement*	PPV	Crystalloid and Colloid	15%	0.56
Feissel et al (5)	2005	20	20	SEPSIS	FC (8 ml/kg)	TTE	CI	Arterial tracing measurement*	PPV	Colloid	15%	0.65
Hofer et al (6)	2005	35	35	CARDIOVASCULAR	FC (10 ml/kg)	TDTP (PiCCO)	SVI	PCA (PiCCO)	PPV. SVV	Colloid	25%	0.6
Lafanechere et al (7)	2006	22	22	SEPSIS	FC (500 ml)	Esophageal doppler	ABF	Arterial tracing measurement*/ Esophageal Doppler	PPV. PLR	Crystalloid	15%	0.45
Monnet et al (8)	2006	71	71	SEPSIS	FC (500 ml)	Esophageal doppler	ABF	Esophageal doppler/Arterial pressure	PLR	Crystalloid	15%	0.54
Monnet et al (8)	2006	30	30	SEPSIS	FC (500 ml)	Esophageal doppler	ABF	Arterial tracing measurement*	PPV	Crystalloid	15%	0.54
Charron et al (9)	2006	21	21	SEPSIS	FC (100 ml)	TEE	SV	Arterial tracing measurement*	PPV	Colloid	15%	0.43
Cannesson et al (10)	2007	25	25	CARDIOVASCULAR	FC (500 ml)	PAC	CI	Arterial tracing measurement*	PPV	Colloid	15%	0.6
Feissel et al (11)	2007	23	23	SEPSIS	FC (8 ml/kg)	TTE	CI	Arterial tracing measurement*	PPV	Colloid	15%	0.64
Wyffels et al (12)	2007	32	32	CARDIOVASCULAR	FC (500 ml)	C-TD	CI	Arterial tracing measurement*	PPV	Colloid	15%	0.625
Maizel et al (13)	2007	34	34	MEDICAL INTENSIVE CARE UNIT	FC (500 ml)	TTE	CO. SV	TTE	PLR	Crystalloid	5%	0.5
Lamia et al (14)	2007	24	24	SEPSIS	FC (500 ml)	TTE	SVI	TTE	PLR	Crystalloid	15%	0.54
Auler et al (15)	2008	59	59	CARDIOVASCULAR	FC (20 ml/kg)	PAC	CI	PCA (computer software)	PPV	Crystalloid	15%	0.66

Study	Year	Sample Size	Number of Fluid Challenges	Setting	Excitation method	Measurement method to assess excitation method	Peri-excitation macrohemodynamic variable	Method Used to Measure Predictor	Predictor Of Fluid Responsiveness evaluated	Type of fluid used	Definition of Fluid Responsiveness	Fluid Responsiveness Rate
Cannesson et al (16)	2008	25	25	CARDIOVASCULAR	FC (500 ml)	PAC	CI	Arterial tracing measurement*/PCA (computer software)	PPV	Colloid	15%	0.64
Huang et al(17)	2008	22	22	SEPSIS	FC (500 ml)	PAC	CI	PCA (PiCCO)	PPV	Colloid	15%	0.45
Derichard et al (18)	2009	11	56	SURGICAL	FC (300 ml)	Esophageal doppler	SVI	Arterial tracing measurement*/PCA (computer software)	PPV	Colloid	10%	0.57
de Wall et al (19)	2009	22	22	CARDIOVASCULAR	FC (10 ml/kg)	TDTP (PiCCO)	SVI	PCA (PiCCO)	PPV/SVV	Colloid	12%	0.5
Cannesson et al (20)	2009	25	25	CARDIOVASCULAR	FC (500 ml)	PAC	CI	Arterial tracing measurement*/PCA (Vigileo)	PPV/SVV	Colloid	15%	0.68
Monge et al (21)	2009	38	38	SEPSIS	FC (500 ml)	NC-PCA (Vigileo)	SVI	Arterial tracing measurement*/PCA (Vigileo)	PPV/SVV	Colloid	15%	0.5
Vistisen et al (22)	2009	23	23	CARDIOVASCULAR	FC (500 ml)	C-TD	CI	Arterial tracing measurement*	PPV	Colloid	15%	0.73
Vallee et al (23)	2009	42	42	SEPSIS	FC (6 ml/kg)	TDTP (PiCCO)	CI	Arterial tracing measurement*	PPV	Colloid	15%	0.38
Lahner et al (24)	2009	20	67	SURGICAL	FC (250 ml)	Esophageal doppler	SVI	PCA (Vigileo)	SVV	Colloid or Crystalloid	10%	0.77
Biais et al (25)	2009	30	30	SURGICAL	FC (500 ml)	TTE	SV	TTE/PCA (Vigileo)	PLR	Crystalloid	15%	0.66
Thiel et al (26)	2009	89	102	SEPSIS	FC (500 ml)	Ultrasonic cardiac output monitor (USCOM technology)	SV	Ultrasonic cardiac output monitor (USCOM technology)	PLR	Crystalloid and Colloid	15%	0.46
Monnet et al (27)	2009	34	34	SEPSIS	FC (500 ml)	TDTP (PiCCO)	CI	Arterial pressure/PCA (PiCCO)	EEOT	Crystalloid	15%	0.67
Muller et al (28)	2010	57	57	SEPSIS	FC (250-500 ml)	PAC and TDTP (PiCCO)	SVI	PCA (computer software)	PPV	Crystalloid and Colloid	15%	0.72
Biais et al (29)	2010	27	27	NEUROSURGICAL	FC (500 ml)	NC-PCA (Vigileo)	CO	Arterial tracing measurement*/PCA (Vigileo)	PPV/SVV	Colloid	15%	0.59

Study	Year	Sample Size	Number of Fluid Challenges	Setting	Excitation method	Measurement method to assess excitation method	Peri-excitation macrohemodynamic variable	Method Used to Measure Predictor	Predictor Of Fluid Responsiveness evaluated	Type of fluid used	Definition of Fluid Responsiveness	Fluid Responsiveness Rate
Zimmermann et al (30)	2010	20	20	SURGICAL	FC (7 ml/kg)	NC-PCA (Vigileo)	SVI	PCA (Vigileo)	SVV	Colloid	15%	0.75
Préau et al (31)	2010	34	34	SEPSIS	FC (500 ml)	TTE	SV	TTE/Arterial pressure/Ultrasonic cardiac output monitor (USCOM technology)	PLR	Colloid	15%	0.41
Lakhal et al (32)	2011	65	65	SEPSIS	FC (500 ml)	PAC and TDTP (PiCCO)	CO	Arterial tracing measurement*	PPV	Colloid	10%	0.4
Geerts et al (33)	2011	24	24	CARDIOVASCULAR	FC (500 ml)	NC-PCA (ModelFlow)	CO	PCA (ModelFlow)	PPV/SVV	Colloid	10%	0.7
Desgranges et al (34)	2011	28	28	CARDIOVASCULAR	FC (500 ml)	PAC	CI	PCA (computer software)	PPV	Colloid	15%	0.67
Machare-Delgado et al (35)	2011	25	25	SEPSIS	FC (500 ml)	TTE	SVI	PCA (Vigileo)	SVV	Crystalloid	10%	0.32
Huang et al (36)	2011	30	30	SEPSIS	FC (250 -500 ml)	Ultrasonic cardiac output monitor (USCOM technology)	SV	Ultrasonic cardiac output monitor (USCOM technology)	PLR	Colloid	5%	0.5
Broch et al (37)	2011	81	81	CARDIOVASCULAR	PLR	TDTP(PiCCO)	SVI	PCA (PiCCO)	PPV, SVV	Na	15%	0,.5
Geerts et al (38)	2011	20	20	CARDIOVASCULAR	PLR	PAC	CO	PCA (LiDCO)	SVV	Na	7%	0.5
Loupec et al (39)	2011	40	40	MEDICAL INTENSIVE CARE UNIT	PLR and FC	ECO TT	CO	Arterial tracing measurement*	PPV	Colloid	15%	0.52
Muller et al (40)	2011	39	39	SEPSIS	FC (500 ml)	TTE	VTI	TTE	MFC	Colloid	15%	0.54
Fellahi et al (41)	2012	25	25	CARDIOVASCULAR	FC (500 ml)	TDTP (PiCCO)	CI	PCA (PiCCO)	PLR	Colloid	15%	0.56
Fellahi et al (42)	2012	25	25	CARDIOVASCULAR	FC (500 ml)	TDTP (PiCCO)	CI	PCA (PiCCO)	PPV/SVV	Colloid	15%	0.84
Khwannimit et al (43)	2012	42	42	SEPSIS	FC (500 ml)	NC-PCA (Vigileo)	SVI	PCA (computer software)/PCA (Vigileo)	PPV/SVV	Colloid	15%	0.57

Study	Year	Sample Size	Number of Fluid Challenges	Setting	Excitation method	Measurement method to assess excitation method	Peri-excitation macrohemodynamic variable	Method Used to Measure Predictor	Predictor Of Fluid Responsiveness evaluated	Type of fluid used	Definition of Fluid Responsiveness	Fluid Responsiveness Rate
Monnet et al (44)	2012	28	28	SEPSIS	FC (500 ml)	TDTP (PiCCO)	CI	PCA (PiCCO)	PPV/PLR/EEOT	Crystalloid	15%	0.53
Monnet et al (44)	2012	26	26	SEPSIS	FC (500 ml)	TDTP (PiCCO)	CI	PCA (PiCCO)	PLR/EEOT	Crystalloid	15%	0.57
Monnet et al (45)	2012	39	39	SEPSIS	FC (500 ml)	TDTP (PiCCO)	CI	Arterial tracing measurement*/PCA (photoplethysmography technology)/PCA (PiCCO)	PPV/SVV/PLR/EEOTT	Crystalloid	15%	0.43
Cecconi et al (46)	2012	31	31	SURGICAL	FC (250 ml)	C-PCA (LiDCO)	SV	PCA (LiDCO)	PPV/SVV	Colloid	15%	0.39
Yazigi et al (47)	2012	60	60	CARDIOVASCULAR	FC (7 ml/kg)	PAC	SVI	Arterial tracing measurement*	PPV	Colloid	15%	0.68
Oliveira-Costa et al (48)	2012	37	37	SEPSIS	FC (500-1000 ml)	PAC	CI	Arterial tracing measurement*	PPV	Crystalloid and Colloid	15%	0.45
Biais et al (49)	2012	35	35	SURGICAL	FC (500 ml)	TTE	SV	Arterial tracing measurement*/PCA (MostCare)	PPV/SVV	Crystalloid	15%	0.54
Fu et al (50)	2012	51	51	SURGICAL	FC (8 ml/kg)	NC-PCA (Vigileo)	SVI	PCA (Vigileo)	SVV	Colloid	10%	0.6
J Li et al (51)	2012	48	157	NEUROSURGICAL	FC (200 ml)	PAC	SV	PCA (Vigileo)	SVV	Crystalloid	10%	0.68
Dong et al (52)	2012	32	32	SEPSIS	FC (500 ml)	C-PCA (PiCCO)	SVI	PCA (PiCCO)	PLR	Colloid	15%	0.68
Monge et al (53)	2012	37	37	SEPSIS	FC (500 ml)	Esophageal doppler	CO	Esophageal doppler/Capnography/Arterial pressure	PLR	Colloid	15%	0.57
Broch et al(54)	2012	92	92	CARDIOVASCULAR	PLR	TDTP(PiCCO)	SVI	PCA (PiCCO)	PPV, SVV	Na	15%	0.57
Monnet et al (55)	2013	35	35	SEPSIS	FC (500 ml)	TDTP (PiCCO)	CI	PCA (PiCCO)	PPV/SVV	Crystalloid	15%	0.42
Monnet et al (56)	2013	40	40	SEPSIS	FC (500 ml)	TDTP (PiCCO)	CI	PCA (PiCCO)/Capnography	PLR	Crystalloid	15%	0.52
Davies et al (57)	2013	21	96	SURGICAL	FC (250 ml)	Esophageal doppler	SV	PCA (LiDCO)	PPV/SVV	Colloid	10%	0.36

Study	Year	Sample Size	Number of Fluid Challenges	Setting	Excitation method	Measurement method to assess excitation method	Peri-excitation macrohemodynamic variable	Method Used to Measure Predictor	Predictor Of Fluid Responsiveness evaluated	Type of fluid used	Definition of Fluid Responsiveness	Fluid Responsiveness Rate
Nordstrom et al (58)	2013	20	86	SURGICAL	FC (200 ml)	Esophageal doppler	SV	PCA (LiDCO)	PPV/SVV	Colloid	10%	0.31
Fischer et al (59)	2013	37	37	CARDIOVASCULAR	FC (500 ml)	TDTP (PiCCO)	CI	PCA (PiCCO)/PCA (photoplethysmography technology)	PPV/SVV	Colloid	15%	0.73
Ishihara et al (60)	2013	43	43	SURGICAL	FC (250 ml)	TDTP (PiCCO)	CI	PCA (PiCCO)	PPV	Colloid	15%	0.5
Drvar et al (61)	2013	46	46	SEPSIS	FC (500 ml)	TDTP (LiDCO)	SVI	PCA (LiDCO)	PPV	Colloid	15%	0.57
Freitas et al (62)	2013	40	40	SEPSIS	FC (7 ml/kg)	C-TD	CO	PCA (computer software)	PPV	Colloid	15%	0.47
Trepte et al(63)	2013	24	72	SURGICAL	FC (300 ml)	TDTP (PiCCO)	CI	PCA (PiCCO)	PPV/SVV	Colloid	10%	0.57
Yang et al (64)	2013	44	44	NEUROSURGICAL	FC (6 ml/kg)	Esophageal doppler	SVI	PCA (computer software)	PPV	Colloid	10%	0.59
Kim et al (65)	2013	33	33	CARDIOVASCULAR	FC (500 ml)	PAC	SVI	PCA (Vigileo)	SVV	Colloid	12%	0.63
Saugel et al (66)	2013	24	24	MEDICAL INTENSIVE CARE UNIT	FC (7 ml/kg)	TDTP (PiCCO)	CI	PCA (PiCCO)	PLR	Crystalloid	15%	0.29
Kupersztych-Hagege et al (67)	2013	48	48	SEPSIS	FC (500 ml)	TDTP (PiCCO)	CI	PCA (PiCCO)	PLR	Crystalloid	15%	0.6
Silva et al(68)	2013	34	34	SEPSIS	PLR	C-PCA(PiCCO)	CI	PCA (PiCCO)	EEOT	Na	10%	0,29
Pei et al (69)	2014	32	32	SURGICAL	FC (250 ml)	NC-PCA (Vigileo)	SVI	PCA (computer software)/PCA (Vigileo)	PPV	Colloid	10%	0.34
Hoiseth et al (70)	2014	23	31	CARDIOVASCULAR	FC (250-500 ml)	Esophageal doppler	SV	Arterial tracing measurement*/PCA (Vigileo)	PPV/SVV	Crystalloid. Colloid and PRBC	15%	0.37
Guarracino et al (71)	2014	50	50	SEPSIS	FC (7 ml/kg)	NC-PCA (MostCare)	CI	PCA (MostCare)	PPV	Crystalloid	15%	0.6
Song et al (72)	2014	40	40	CARDIOVASCULAR	FC (6 ml/kg)	C-TD	SVI	PCA (computer software)	PPV	Colloid	15%	0.57
Shim et al (73)	2014	34	34	CARDIOVASCULAR	FC (6 ml/kg)	C-TD	SVI	PCA (computer software)	PPV	Colloid	12%	0.5

Study	Year	Sample Size	Number of Fluid Challenges	Setting	Excitation method	Measurement method to assess excitation method	Peri-excitation macrohemodynamic variable	Method Used to Measure Predictor	Predictor Of Fluid Responsiveness evaluated	Type of fluid used	Definition of Fluid Responsiveness	Fluid Responsiveness Rate
Fisher et al (74)	2014	50	50	CARDIOVASCULAR	FC (500 ml)	TDTP (PiCCO)	CI	PCA (PiCCO)	PPV	Colloid	15%	0.82
Kurts et al (75)	2014	10	57	NEUROSURGICAL	FC (250 ml)	C-PCA (PiCCO)	CI	PCA (PiCCO)	SVV	Colloid	10%	0.47
Guinot et al (76)	2014	42	42	SURGICAL	FC (500 ml)	Esophageal doppler	SV	Esophageal doppler	SVV	Crystalloid	15%	0.67
Kang et al (77)	2014	54	54	CARDIOVASCULAR	FC (250 ml)	IMPEDANCE	CO	Bioimpedance technique	SVV	Colloid	7%	0.5
Guinot et al (78)	2014	42	42	SURGICAL	FC (500 ml)	Esophageal doppler	SV	Esophageal doppler/TTE	EEOT	Crystalloid	15%	0.67
Wu et al (79)	2014	50	50	SEPSIS	FC (500 ml)	TTE	SV	TTE	MFC	Crystalloid	15%	0.54
Messina et al (80)	2015	27	27	SEPSIS	FC (500 ml)	NC-PCA (Mostcare)	CI	PCA (MostCare)	PPV	Crystalloid	15%	0.33
Zhao et al (81)	2015	25	25	SURGICAL	FC (250 ml)	NC-PCA (Vigileo)	SVI	PCA (computer software)/PCA (Vigileo)	PPV/SVV	Colloid	10%	0.48
Ibarra-Estrada et al (82)	2015	19	59	SEPSIS	FC (7 ml/kg)	TDTP (PiCCO)	SVI	PCA (computer software)/PCA (PiCCO)	PPV/SVV/PLR	Crystalloid	15%	0.51
Angappan et al (83)	2015	45	45	SEPSIS	FC (500 ml)	NC-PCA (Vigileo)	CI	PCA (Vigileo)	SVV	Colloid	15%	0.64
Airapetian et al (84)	2015	59	59	MEDICAL INTENSIVE CARE UNIT	FC (500 ml)	TTE	CO	Abdominal ultrasonography /TTE	PLR	Crystalloid	10%	0.49
Guinot et al (85)	2015	73	73	SURGICAL	FC (500 ml)	IMPEDANCE	SV	IMPEDANCE	MFC	Crystalloid	15%	0.37
Mallat et al (86)	2015	49	49	SEPSIS	FC (500 ml)	TDTP(PiCCO)	CI	PCA (PiCCO)	MFC	Colloid	15%	0.45
Theerawit et aL (87)	2016	29	29	SEPSIS	FC (500-1000 ml)	NC-PCA (Vigileo)	CO	PCA (Vigileo)	SVV	Crystalloid and Colloid	15%	0.55
Lee et al (88)	2016	40	40	SURGICAL	FC (6 ml/kg)	TTE	SVI	PCA (computer software)/PCA (Vigileo)	PPV/SVV	Colloid	15%	0.65
Cherpanath et al (89)	2016	22	22	CARDIOVASCULAR	FC (500 ml)	NC-PCA (ModelFlow)	CO	PCA (computer software)	PPV/SVV	Colloid	15%	0.86
Zhang et al (90)	2016	40	40	SURGICAL	FC (7 ml/kg)	NC-PCA (Vigileo)	SVI	PCA (Vigileo)	SVV	Colloid	15%	0.65

Study	Year	Sample Size	Number of Fluid Challenges	Setting	Excitation method	Measurement method to assess excitation method	Peri-excitation macrohemodynamic variable	Method Used to Measure Predictor	Predictor Of Fluid Responsiveness evaluated	Type of fluid used	Definition of Fluid Responsiveness	Fluid Responsiveness Rate
Fisher et al (91)	2016	78	78	CARDIOVASCULAR	FC (500 ml)	TDTP (PiCCO)	CI	PCA (PiCCO)	PLR	Colloid	15%	0.71
Kim et al (92)	2016	43	43	CARDIOVASCULAR	FC (300 ml)	C-TD	SVI	C-TD	PLR	Colloid	10%	0.35
de Oliveira et al (93)	2016	20	20	NEUROSURGICAL	FC (500 ml)	ECO TT	VTI	PCA (computer software)	PPV	Crystalloid	15%	0.45
Sobczyk et al (94)	2016	35	35	CARDIOVASCULAR	FC (250 ml)	TTE	CO	TTE	PLR	Crystalloid	15%	0.68
Liu et al (95)	2016	96	96	SEPSIS	FC (500 ml)	TDTP (PiCCO)	CO	PCA (PiCCO)	PPV	Crystalloid	15%	0.54
Vistisen et al (96)	2016	41	41	CARDIOVASCULAR	FC (500 ml)	C-TD	SV	Arterial tracing measurement*	PPV	Crystalloid and Colloid	15%	0.41
Lagreze et al (97)	2016	40	40	SURGICAL	FC (500 ml)	Esophageal doppler	CO	Capnography	MFC	Colloid	15%	0.38
Kridge et al (98)	2016	33	33	SEPSIS	FC (250 ml)	NC-PCA(Vigileo)	CO	PCA (Vigileo)	PLR	Colloid	15%	0.3
Myatra et al (99)	2017	20	30	SEPSIS	FC (7 ml/kg)	TDTP (PiCCO)	CI	PCA (computer software)/PCA (PiCCO)	PPV/SVV/EEOT/VTC	Crystalloid	15%	0.53
Yonis et al (100)	2017	33	33	SEPSIS	FC (500 ml)	TDTP (PiCCO)	CI	PCA (PiCCO)	PPV/EEOTT	Crystalloid	15%	0.45
Renner et al (101)	2017	39	39	SURGICAL	FC (500 ml)	TDTP (PiCCO)	SVI	PCA (PiCCO)/PCA (photoplethysmography technology)	PPV	Crystalloid	15%	0.61
Min et al (102)	2017	40	40	NEUROSURGICAL	FC (6 ml/kg)	NC-PCA (Vigileo)	CI	PCA (computer software)/Arterial tracing measurement*/PCA (Vigileo)	PPV/SVV	Colloid	12%	0.47
Blais et al (103)	2017	41	41	NEUROSURGICAL	FC (250 ml)	NC-PCA (Pulsioflex/ProAQT)	SVI	PCA (Pulsioflex system) /Others/Arterial pressure	PPV/EEOTT	Crystalloid	10%	0.48
Biais et al (104)	2017	88	44	NEUROSURGICAL	FC (250 ml)	NC-PCA(PulsioFlex/ProAQT)	SVI	PCA (Pulsioflex)	MFC	Crystalloid	10%	0.31
Messina et al (105)	2017	46	46	SURGICAL	FC (500 ml)	NC-PCA (MostCare)	CI	PCA (MostCare)	PPV	Crystalloid	15%	0.41
Lu et al (106)	2017	49	49	SEPSIS	FC (200 ml)	C-PCA (PiCCO)	CI	PCA (PiCCO)	SVV	Crystalloid	10%	0.55

Study	Year	Sample Size	Number of Fluid Challenges	Setting	Excitation method	Measurement method to assess excitation method	Peri-excitation macrohemodynamic variable	Method Used to Measure Predictor	Predictor Of Fluid Responsiveness evaluated	Type of fluid used	Definition of Fluid Responsiveness	Fluid Responsiveness Rate
El Hadouti et al (107)	2017	41	41	SURGICAL	FC (500 ml)	TTE	SV	TTE	PLR	Crystalloid	15%	0.54
Jozwiak et al (108)	2017	30	30	SEPSIS	FC (500 ml)	TDTP (PiCCO)	CI	PCA (PiCCO)/TTE	EEOT	Crystalloid	15%	0.5
Renner et al (109)	2017	46	46	SURGICAL	PLR	TD	SVI	PCA (PiCCO)	PLR	Na	15%	0.45
Xu et al (110)	2017	34	34	SEPSIS	FC (250 ml)	IMPEDANCE	SV	IMPEDANCE	SVV	Crystalloid	10%	0.41
Guo-Guang Ma et al (111)	2018	70	70	CARDIOVASCULAR	FC (500 ml)	NC-PCA (Vigileo)	SV	PCA (Vigileo)	SVV/PLR	Colloid	10%	0.5
Wu et al (112)	2018	62	62	SEPSIS	FC (250 ml)	TTE	SV	TTE/Ultrasonic cardiac output monitor (USCOM technology)	PLR	Crystalloid	15%	0.45
Si et al (113)	2018	40	40	SEPSIS	FC (250 ml)	TDTP (PiCCO)	SV	PCA (PiCCO)/Arterial pressure	PLR	Colloid	15%	0.65
Georges et al (114)	2018	50	50	NEUROLOGICAL	FC (500 ml)	ECO TT	CO	TTE	EEOT	Crystalloid	15%	0.56
Joosten et al (115)	2019	57	57	CARDIOVASCULAR	FC (5 ml/kg)	TDTP (PiCCO)	CO	Calculated by application/PCA (computer software)	PPV	Colloid	10%	0.46
Ali et al (116)	2019	88	88	NEUROSURGICAL	FC (500 ml)	NC-PCA (Vigileo)	SVI	PCA (computer software)	PPV	Crystalloid	15%	0.44
He et al (117)	2019	79	79	SURGICAL	FC (6 ml/kg)	TTE	VTI	PCA (computer software)	PPV	Colloid	15%	0.48
Ali et al (118)	2019	33	33	NEUROSURGICAL	FC (500 ml)	NC-PCA (Vigileo)	SVI	PCA (computer software)/PCA (Vigileo)	PPV/SVV	Crystalloid	15%	0.45
Xu et al (119)	2019	75	75	CARDIOVASCULAR	FC (6 ml/kg)	TEE	VTI	PCA (Vigileo)/TEE/Arterial pressure	SVV/EEOT	Crystalloid	15%	0.48
Trifi et al (120)	2019	26	30	SEPSIS	FC (500 ml)	TTE	SV	TTE	PLR	Crystalloid	15%	0.63
Beurton et al (121)	2019	30	30	SEPSIS	FC (500 ml)	TDTP (PiCCO)	CI	PCA (PiCCO)	PLR	Crystalloid	15%	0.5

Study	Year	Sample Size	Number of Fluid Challenges	Setting	Excitation method	Measurement method to assess excitation method	Peri-excitation macrohemodynamic variable	Method Used to Measure Predictor	Predictor Of Fluid Responsiveness evaluated	Type of fluid used	Definition of Fluid Responsiveness	Fluid Responsiveness Rate
Depret el at (122)	2019	28	28	SEPSIS	FC (500 ml)	TDTP (PiCCO)	CI	PCA (PiCCO)/Esophageal doppler	EEOT	Crystalloid	15%	0.5
Messina et al (123)	2019	40	40	NEUROQX	FC (250 ml)	NC-PCA (Mostcare)	SVI	Others	EEOT/VTC	Crystalloid	10%	0.52
Fot et al (124)	2019	33	33	CARDIOVASCULAR	FC (7 ml/kg)	TDTP(PiCCO)	CI	PCA (PiCCO)	MFC	Crystalloid	15%	0.43
Beurton et al (125)	2019	72	72	SEPSIS	PLR	C-PCA(PiCCO)	CI	Others	PLR	Na	10%	0.47
Jun et al (126)	2019	38	38	SURGICAL	FC (6 ml/kg)	Esophageal doppler	SVI	Esophageal doppler/Others	VTC	Colloid	15%	0.64
Lee et al (127)	2020	50	50	NEUROSURGICAL	FC (500 ml)	NC-PCA (Vigileo)	SV	PCA (computer software)/PCA (Vigileo)	PPV/SVV	Colloid	15%	0.6
Wang et al (128)	2020	44	44	SEPSIS	FC (500 ml)	TDTP (PiCCO)	CI	PCA (PiCCO)	PPV/SVV	Crystalloid	15%	0.54
Bapteste et al (129)	2020	31	31	NEUROSURGICAL	FC (250 ml)	Esophageal doppler	SV	PCA (computer software)	PPV	Colloid	10%	0.42
Bubenek et al (130)	2020	40	266	SURGICAL	FC (500 ml)	NC-PCA (Vigileo)	CI	PCA (Vigileo)	SVV	Colloid	11%	0.91
Hou et al (131)	2020	102	102	SEPSIS	FC (250 ml)	NC-PCA (Vigileo)	SV	Arterial pressure	PLR	Colloid	15%	0.52
Weil et al (132)	2020	46	115	SURGICAL	FC (250 ml)	NC-PCA (PulsioFlex/ProAQT)	CI	Others	EE0	Colloid	5%	0.38
Messina et al (133)	2020	40	40	NEUROSURGICAL	FC (250 ml)	NC-PCA(MostCare)	SVI	PCA(MostCare)	VTC	Crystalloid	10%	0.47
Hamzaoui et al (134)	2021	54	54	MEDICAL INTENSIVE CARE UNIT	PLR	ECO TT	VTI	TTE	VTC, PLR	Na	12%	0.4
Giraud et al (135)	2021	33	33	CARDIOVASCULAR	PLR	ECO TT	VTI	Others	PLR	Na	15%	0.45
Kaur et al (136)	2021	67	67	MEDICAL INTENSIVE CARE UNIT	FC (10 ml/kg)	NC-PCA (Vigileo)	CO	PCA (Vigileo)	SVV	Crystalloid	15%	0.67

Study	Year	Sample Size	Number of Fluid Challenges	Setting	Excitation method	Measurement method to assess excitation method	Peri-excitation macrohemodynamic variable	Method Used to Measure Predictor	Predictor Of Fluid Responsiveness evaluated	Type of fluid used	Definition of Fluid Responsiveness	Fluid Responsiveness Rate
Bataille et al (137)	2021	100	100	SEPSIS	FC (500 ml)	TTE	SV	TTE	PLR	Crystalloid	15%	0.5
Lizuka et al (138)	2021	41	41	SURGICAL	FC (250 ml)	NC-PCA (Vigileo)	SV	Others/PCA (Vigileo)	EEOT	Crystalloid	10%	0.39
Messina et al (139)	2021	103	103	SURGICAL	FC (4 ml/kg)	NC-PCA (MostCare)	SVI	PCA (MostCare)	PPV, SVV, EEOT, MFC	Crystalloid	10%	0.51
Watanabe et al(140)	2021	30	30	NEUROSURGICAL	FC (250 ml)	NC-PCA(Vigileo)	SV	Arterial tracing measurement*	PPV, SVV	Colloid	10%	0.43
Chen et al (141)	2021	27	27	SEPSIS	FC (500 ml)	TDTP(PiCCO)	CI	PCA (PiCCO)	PPV, SVV	Colloid	15%	0.59
Taccheri et al (142)	2021	30	30	SEPSIS	PLR	C-PCA(PiCCO)	CI	PCA (PiCCO)	VTC, PLR	Na	10%	0.5
Elsayed et al (143)	2021	46	46	SEPSIS	FC (4 ml/kg)	TTE	CO	TTE	VTC, PLR	Crystalloid	15%	0.34
Shi et al (144)	2022	84	58	SEPSIS	Trendelenburg and EEOT	TDTP(PiCCO)	CI	PCA (PiCCO)	EEOT, VTC, PPV	Na	5% or 8%	0.5
Shi et al (145)	2022	35	35	SEPSIS	PLR	C-PCA(PiCCO)	CI or VTI	Arterial pressure	PLR	Na	10% or 12%	0.4
Abdullah et al(146)	2022	55	55	MEDICAL INTENSIVE CARE UNIT	FC (500 ml)	NC-PCA(Vigileo)	SVI	PCA (Vigileo)	SVV	Crystalloid	15%	0.46
Xu et al (147)	2022	76	76	SEPSIS	FC (6 ml/kg)	TDTP(PICCO)	CI	PCA (PICCO)	VTC	Crystalloid	15%	0.4
Mallat et al (148)	2022	270	270	MEDICAL INTENSIVE CARE UNIT	FC (4 ml/kg)	TTE and TDTP(PiCCO)	CI	PCA (PiCCO) and TTE	PLR	Crystalloid	15%	0.6
Botros et al (149)	2023	48	48	SURGICAL	FC (6 ml/kg)	Esophageal doppler	SVI	Others	VTC	Crystalloid	10%	0.41

Table S1. General characteristics of selected studies. Values are expressed as pooled values (95% confidence interval). ABF, aortic blood flow; C-TD, continuous thermodilution; C-PCA, calibrated pulse contour analysis; CO, cardiac output; CI, cardiac index; EEOT, end-expiratory occlusion test; FC; fluid challenge; NC-PCA, non-calibrated pulse contour analysis; PAC, pulmonary artery catheter; PLR, passive leg raising; PPV, pulse pressure variation; PRBC, packed red blood cells; SV, stroke volume; SVI, stroke volume index; SVV, stroke volume variation; TD, thermodilution; TEE, transesophageal echocardiography; TPTD, transpulmonary thermodilution; TTE, transthoracic echocardiography; VTI, velocity time integral. *The arterial tracing measurement elucidates the manually performed measurement of Pulse Pressure Variation.

Study	Year	Number total of patients	True positive	Fluid responders	True negative	Non-fluid responders	Cut-off	Sensitivity	Specificity	AUC
Michard et al (1)	2000	40	15	16	21	22	13	0.94	0.96	0.98±0.03
Kramer et al (3)	2004	21	6	6	14	15	11	1	0.93	0.99
De Backer et al (4)	2005	27	13	15	11	12	12.8	0.88	0.89	0.89±0.07
De Backer et al (4)	2005	33	12	18	10	15	8	0.66	0.65	0.71±0.09
Feissel et al (5)	2005	20	11	13	7	7	17	0.85	1	0.96±0.03
Hofer et al (6)	2005	35	15	21	10	14	13.5	0.72	0.72	0.80(0.67-0.94)
Lafanechere et al (7)	2006	22	7	10	11	12	12	0.7	0.92	0.78
Charron et al (9)	2006	21	8	9	10	12	10	0.89	0.83	0.95(0.85-1.00)
Monnet et al (8)	2006	30	14	16	13	14	12	0.88	0.93	0.91±0.05
Cannesson et al (10)	2007	25	12	15	9	10	11	0.8	0.9	0.84±0.08
Feissel et al (11)	2007	28	18	18	7	10	12	1	0.7	0.94±0.05
Wyffels et al (12)	2007	32	19	20	11	12	11.8	0.95	0.91	0.94(0.79-0.99)
Auler et al (15)	2008	59	38	39	19	20	12	0.97	0.95	0.98±0.01
Cannesson et al (16)	2008	25	14	16	8	9	12.5	0.87	0.89	0.93±0.04
Cannesson et al (16)	2008	25	14	16	8	9	10.5	0.87	0.89	0.94±0.04
Huang et al(17)	2008	22	7	10	12	12	11.8	0.68	1	0.76
Derichard et al (18)	2009	56	28	32	22	24	13	0.88	0.92	0.96(0.70-1.00)
Derichard et al (18)	2009	56	29	32	22	24	13	0.89	0.91	0.96(0.72-1.00)
de Wall et al (19)	2009	22	7	11	11	11	10	0.64	1	0.88±0.0.07
Cannesson et al (20)	2009	25	15	17	7	8	10	0.88	0.87	0.85±0.08
Monge et al (21)	2009	38	18	19	18	19	10	0.95	0.95	0.97±0.03
Vistisen et al (22)	2009	23	16	17	5	6	6.5	0.94	0.83	NR
Vallee et al (23)	2009	42	5	16	19	26	15	0.32	0.75	0.62(0.45-0.80)

Study	Year	Number total of patients	True positive	Fluid responders	True negative	Non-fluid responders	Cut-off	Sensitivity	Specificity	AUC
Vallee et al (23)	2009	42	16	24	14	18	15	0.65	0.77	0.75(0.60-0.89)
Muller et al (28)	2010	57	25	41	15	16	7	0.61	0.94	0.77(0.65-0.90)
Biais et al (29)	2010	27	14	16	9	11	11	0.88	0.82	0.94(0.78-0.99)
Biais et al (29)	2010	27	17	17	8	10	15	1	0.8	0.95(0.80-0.99)
Lakhal et al (32)	2011	65	19	26	33	39	6.5	0.73	0.85	0.75(0.62-0.85)
Geerts et al (33)	2011	24	13	17	5	7	9	0.77	0.71	0.84(0.64-0.96)
Broch et al (37)	2011	81	36	45	27	36	8	0.79	0.76	0.83(0.74-0.96)
Loupec et al	2011	40	21	21	18	19	9	1	0.94	0.97(0.86-0.99)
Desgranges et al (34)	2011	28	14	19	8	9	8	0.74	0.89	0.83±0.08
Fellahi et al (42)	2012	25	17	21	3	4	6.5	0.81	0.75	0.86(0.67-1.06)
Khwannimit et al (43)	2012	42	20	24	15	18	8	0.83	0.83	0.91(0.82-1.00)
Monnet et al (45)	2012	39	15	17	20	22	10	0.88	0.91	0.89(0.77-1.01)
Monnet et al (45)	2012	39	14	17	20	22	11	0.82	0.91	0.89(0.78-1.01)
Monnet et al (44)	2012	26	13	15	11	11	12	0.85	1	0.98±0.03
Monnet et al (44)	2012	28	15	15	4	13	4	1	0.31	0.69±0.10
Cecconi et al (46)	2012	31	17	20	8	11	13	0.83	0.74	0.87(0.76-0.99)
Yazigi et al (47)	2012	60	33	41	14	19	11.5	0.8	0.74	0.85(0.75-0.94)
Oliveira-Costa et al (48)	2012	37	9	17	19	20	10	0.53	0.95	0.74(0.56-0.90)
Biais et al (49)	2012	35	17	19	14	16	10	0.89	0.88	0.95(0.82-0.99)
Broch et al	2012	92	37	53	31	39	11	0.7	0.79	0.82(0.73-0.91)
Monnet et al (55)	2013	35	14	15	19	20		0.93	0.95	0.93±0.06
Davies et al (57)	2013	96	30	35	21	61	12	0.84	0.25	0.61(0.46-0.76)
Nordstrom et al (58)	2013	86	21	27	27	59	8.5	0.79	0.45	0.66(0.52-0.79)
Fischer et al (59)	2013	37	12	27	10	10	16	0.44	1	0.73(0.57-0.86)
Fischer et al (59)	2013	37	13	27	8	10	19	0.48	0.8	0.57(0.40-0.73)

Study	Year	Number total of patients	True positive	Fluid responders	True negative	Non-fluid responders	Cut-off	Sensitivity	Specificity	AUC
Ishihara et al (60)	2013	43	11	22	14	20	8.5	0.5	0.71	0.65(0.48-0.82)
Drvar et al (61	2013	46	26	26	20	20	8.5	1	1	1(0.92-1)
Freitas et al (62)	2013	40	17	19	10	11	6.5	0.89	0.9	0.91(0.82-1)
Trepte et al(63)	2013	72	25	41	25	31	6.5	0.61	0.8	0.70(0.21-0.85)
Yang et al (64)	2013	44	19	26	17	18	15	0.73	0.94	0.93(0.87-0.99)
Yang et al (64)	2013	44	33	34	9	10	14	0.97	0.9	0.96(0.91-1.00)
Pei et al (69)	2014	32	7	11	15	21	10	0.63	0.71	0.71(0.52-0.85)
Hoiseth et al (70)	2014	27	10	10	16	17	13	1	0.94	0.95(0.80-1.00)
Guarracino et al (71)	2014	50	29	30	11	20	12.5	0.96	0.55	0.85(0.72-0.93)
Song et al (72)	2014	40	17	23	12	17	13	0.74	0.71	0.75(0.59-0.91)
Shim et al (73)	2014	34	13	17	12	17	12	0.77	0.71	0.82(0.660.93)
Fisher et al (74)	2014	50	34	41	7	9	10	0.83	0.78	0.87(0.75-0.95)
Messina et al (80)	2015	27	8	9	13	18	10	0.89	0.72	0.86(0.68-0.96)
Zhao et al (81)	2015	25	11	12	9	13	8	0.91	0.69	0.87(0.68-0.97))
Ibarra-Estrada et al (82)	2015	59	15	30	15	19	14	0.5	0.79	0.63(0.49-0.75)
Vistisen et al (96)	2016	41	11	17	18	24	12	0.65	0.75	0.57(0.39-0.75)
Liu et al (95)	2016	96	35	52	37	44	10	0.67	0.84	0.78(0.69-0.86)
de Oliveira et al (93)	2016	20	8	9	11	11	12.4	0.88	1	0.92±0.08
Theerawit et aL (87)	2016	29	16	16	11	13	13.8	1	0.84	0.90(0.78-1.00)
Lee et al (88)	2016	40	22	26	12	14	15	0.86	0.85	0.81(0.77-10.00)
Cherpanath et al (89)	2016	22	18	19	3	3	8	0.95	1	0.95(0.76-0.99)
Myatra et al (99)	2017	30	12	16	14	14	11.5	0.75	1	0.91(0.81-1.00)
Yonis et al (100)	2017	19	3	9	8	10	10	0.33	0.8	0.49(0.21-0.77)
Yonis et al (100)	2017	19	7	9	4	10	9	0.78	0.4	0.52(0.24-0.80)

Study	Year	Number total of patients	True positive	Fluid responders	True negative	Non-fluid responders	Cut-off	Sensitivity	Specificity	AUC
Renner et al (101)	2017	39	21	24	15	15	13	0.87	1	0.97(0.86-0.97)
Renner et al (101)	2017	39	22	24	14	15	13	0.91	0.93	0.97(0.93-1.01)
Min et al (102)	2017	40	12	19	17	21	12	0.63	0.82	0.76(0.6-0.88)
Min et al (102)	2017	40	18	19	12	21	10	0.94	0.58	0.84(0.69-0.94)
Biais et al (104)	2017	88	15	28	41	60	10	0.54	0.68	0.65(0.53-0.78)
Blais et al (103)	2017	41	12	20	18	21	9	0.6	0.86	0.75(0.60-0.90)
Messina et al (105)	2017	46	15	19	22	26	12.7	0.8	0.85	0.80(0.66-0.94)
Joosten et al (115)	2019	57	19	26	23	31	8.6	0.73	0.74	0.73(0.60-0.84)
Joosten et al (115)	2019	57	16	26	23	31	9.5	0.62	0.74	0.68(0.54-0.80)
Ali et al (116)	2019	88	35	39	46	49	12	0.9	0.94	0.93(0.87-0.99)
Ali et al (116)	2019	88	24	39	46	49	12	0.61	0.93	0.79(0.69-0.87)
He et al (117)	2019	79	21	38	34	41	8.5	0.55	0.83	0.79(0.70-0.89)
He et al (117)	2019	79	29	38	38	41	12.5	0.76	0.93	0.91(0.85-0.98)
He et al (117)	2019	79	33	38	36	41	15.5	0.87	0.9	0.93(0.88-0.99)
Ali et al (118)	2019	33	14	15	8	18	10	0.93	0.44	0.68(0.49-0.83)
Jun et al (126)	2019	38	13	24	11	14	6	0.54	0.79	0.69(0.52-0.83)
Jun et al (126)	2019	38	18	24	11	14	7	0.79	0.79	0.85(0.70-0.95)
Lee et al (127)	2020	50	21	30	12	20	8	0.7	0.6	0.71(0.57-0.86)
Wang et al (128)	2020	44	20	24	15	20	25.8	0.83	0.75	0.84(0.72-0.95)
Bapteste et al (129)	2020	31	11	13	15	18	8	0.83	0.83	0.87(0.72-1.00)
Chen et al (141)	2021	27	16	16	6	11	3.2	1	0.54	0.81(0.61-0.93)
Chen et al (141)	2021	27	12	16	7	11	3.2	0.75	0.63	0.67(0.46-0.84)
Chen et al (141)	2021	27	12	16	10	11	3.2	0.75	0.91	0.85(0.66-0.96)
Watanabe et al(140)	2021	30	11	13	6	17	10	0.84	0.35	0.55(0.35-0.73)
Shi et al (144)	2021	84	31	42	33	42	6.5	0.74	0.79	0.85(0.77-0.92)

Study	Year	Number total of patients	True positive	Fluid responders	True negative	Non-fluid responders	Cut-off	Sensitivity	Specificity	AUC
Messina et al (139)	2021	103	29	53	45	50	10.8	0.54	0.9	0.77(0.67-0.84)
Xu et al (147)	2021	76	22	31	29	45	7	0.71	0.64	0.69(0.57-0.79)
Xu et al (147)	2021	76	25	31	38	45	11	0.8	0.84	0.90(0.81-0.96)
Elsayed et al (143)	2021	46	14	16	25	30	10.5	0.87	0.83	0.87(0.76-0.97)

Table S2. Operative performance of pulse pressure variation for prediction of fluid responsiveness from included studies. Values are expressed as pooled values (95% confidence interval) or median (IQR). AUC, area under curve reported by each study; NE, not reported.

Study	Year	Number total of patients	True positive	Fluid responders	True negative	Non-fluid responders	Cut-off	Sensitivity	Specificity	AUC
Berkenstadt et al (2)	2001	140	55	70	65	70	9.5	0.78	0.932	0.87(0.80-0.90)
Hofer et al (6)	2005	35	16	21	10	14	12.5	0.74	0.71	0.82(0.67-0.96)
de Wall et al (19)	2009	22	11	11	9	11	8	1	0.78	0.91±0.07
Lahner et al (24)	2009	26	13	20	4	6	8.5	0.65	0.67	0.58(0.23-0.82)
Lahner et al (24)	2009	41	27	32	2	9	8.5	0.85	0.25	0.44(0.23-0.70)
Cannesson et al (20)	2009	25	14	17	7	8	10	0.82	0.88	0.87±0.08
Monge et al (21)	2009	38	15	19	17	19	11	0.79	0.89	0.89±0.06
Zimmermann et al (30)	2010	20	15	15	4	5	11	1	0.8	0.99
Biais et al (29)	2010	27	14	16	10	11	9	0.88	0.91	0.93(0.76-0.99)
Biais et al (29)	2010	27	16	17	8	10	14	0.94	0.8	0.93(0.77-0.99)
Machare-Delgado et al (35)	2011	25	4	8	11	17	12	0.5	0.65	0.57
Geerts et al (33)	2011	24	14	17	6	7	8.8	0.82	0.86	0.74(0.54-0.90)
Geerts et al (38)	2011	20	7	10	10	10	7.3	0.70	1	0.90(0.76-1.00)
Broch et al (37)	2011	81	29	45	27	36	12	0.64	0.75	0.72(0.61-0.88)
Fellahi et al (41)	2012	25	17	21	3	4	11	0.81	0.75	0.89(0.74-1.04)
Khwannimit et al (43)	2012	42	22	24	15	18	10	0.91	0.83	0.92(0.83-1.00)
Cecconi et al (46)	2012	31	15	20	9	11	12.5	0.75	0.83	0.84(0.71-0.96)
Monnet et al (45)	2012	39	13	17	18	22	14	0.76	0.82	0.84(0.71-0.97)
Fu et al (50)	2012	51	27	31	17	20	12.5	0.87	0.83	0.86(0.76-0.96)
J Li et al (51)	2012	157	88	108	41	49	11.5	0.81	0.83	0.88±0.03
Biais et al (49)	2012	35	12	19	11	16	12.6	0.63	0.69	0.60(0.43-0.76)
Broch et al(54)	2012	92	34	53	30	39	11	0.64	0.76	0.77(0.61-0.87)
Davies et al (57)	2013	96	30	35	30	61	10	0.85	0.5	0.64(0.52-0.78)
Davies et al (57)	2013	96	28	35	23	61	10	0.8	0.38	0.57(0.43-0.72)
Fischer et al (59)	2013	37	8	27	9	10	16	0.3	0.9	0.50(0.33-0.67)

Study	Year	Number total of patients	True positive	Fluid responders	True negative	Non-fluid responders	Cut-off	Sensitivity	Specificity	AUC
Ishihara et al (60)	2013	43	10	22	16	20	10.5	0.45	0.81	0.60(0.43-0.78)
Kim et al (65)	2013	33	16	21	8	12	13	0.76	0.67	0.80(0.65-0.95)
Nordstrom et al (58)	2013	86	21	27	37	59	8.5	0.79	0.63	0.66(0.52-0.79)
Trepte et al(63)	2013	72	26	41	23	31	9.9	0.63	0.74	0.72(0.21-0.85)
Monnet et al (55)	2013	35	14	15	18	20	10	0.93	0.9	0.89±0.07
Drvar et al (61	2013	46	25	26	20	20	10	0.96	1	0.96(0.85-0.99)
Kim et al (65)	2013	33	16	21	8	12	13	0.76	0.67	0.80(0.65-0.95)
Pei et al (69)	2014	32	5	11	18	21	10	0.45	0.85	0.69(0.51-0.84)
Hoiseth et al (70)	2014	31	10	12	17	19	12	0.83	0.89	0.90(0.74-0.98)
Kurts et al (75)	2014	57	20	27	24	30	9	0.75	0.8	0.86(0.75-0.96)
Guinot et al (78)	2014	42	26	28	12	14	13	0.93	0.86	0.89(0.76-0.97)
Kang et at (77)	2014	54	24	27	24	27	13.5	0.88	0.88	0.90(0.80-0.99)
Kang et at (77)	2014	54	25	27	23	27	13.5	0.92	0.84	0.93(0.83-1.00)
Kang et at (77)	2014	54	25	27	25	27	13.5	0.92	0.92	0.94(0.86-1.00)
Kang et at (77)	2014	54	23	27	24	27	13.5	0.84	0.88	0.90(0.80-0.99)
Kang et at (77)	2014	54	25	27	23	27	13.5	0.92	0.84	0.93(0.83-1.00)
Kang et at (77)	2014	54	25	27	25	27	13.5	0.92	0.92	0.94(0.86-1.00)
Zhao et al (81)	2015	25	12	12	12	13	10	1	0.92	0.95(0.78-0.99)
Ibarra-Estrada et al (82)	2015	59	23	30	13	19	16	0.76	0.68	0.72(0.59-0.83)
Angappan et al (83)	2015	45	23	29	14	16	13	0.78	0.89	0.71(0.56-0.84)
Zhang et al (90)	2016	40	22	26	13	14	15.5	0.84	0.92	0.93(0.85-1.00)
Cherpanath et al (89)	2016	22	17	19	3	3	9	0.89	1	0.95(0.76-0.99)
Theerawit et aL (87)	2016	29	13	16	10	13	12.5	0.81	0.76	0.81(0.64-0.98)
Lee et al (88)	2016	40	24	26	8	14	12	0.92	0.57	0.83(0.65-0.94)
Min et al (102)	2017	40	14	19	14	21	12	0.74	0.67	0.78(0.62-0.90)

Study	Year	Number total of patients	True positive	Fluid responders	True negative	Non-fluid responders	Cut-off	Sensitivity	Specificity	AUC
Min et al (102)	2017	40	13	19	17	21	12	0.68	0.81	0.79(0.63-0.90)
Myatra et al (99)	2017	30	12	16	13	14	10.5	0.75	0.93	0.92(0.82-1.00)
Lu et al (106)	2017	49	20	27	19	22	11.5	0.75	0.85	0.85(0.72-0.96)
Guo-Guang Ma et al (111)	2018	70	32	35	33	35	12	0.91	0.94	0.97(0.89-0.99)
Jun et al (126)	2019	38	16	24	11	14	5	0.67	0.79	0.69(0.52-0.83)
Xu et al (119)	2019	75	26	36	25	39	12	0.72	0.64	0.75(0.64-0.86)
Ali et al (118)	2019	33	13	15	9	18	10	0.87	0.5	0.70(0.52-0.85)
Lee et al (127)	2020	50	26	30	12	20	5	0.87	0.6	0.72(0.57-0.87)
Wang et al (128)	2020	44	16	24	19	20	9	0.66	0.95	0.80(0.66-0.94)
Bubenek et al (130)	2020	266	190	243	23	23	8	0.78	1	0.89(0.85-0.93)
Kaur et al (136)	2021	67	28	45	21	22	9	0.62	0.81	NR
Chen et al (141)	2021	27	15	16	6	11	6	1	0.54	0.75(0.55-0.90)
Chen et al (141)	2021	27	8	16	11	11	6	0.5	1	0.79(0.59-0.92)
Chen et al (141)	2021	27	10	16	11	11	6	0.62	1	0.76(0.55-0.90)
Watanabe et al(140)	2021	30	5	13	14	17	6.29	0.38	0.82	0.61(0.41-0.78)
Messina et al (139)	2021	103	26	53	42	50	7	0.49	0.84	0.71(0.61-0.80)
Abdullah et al(146)	2022	55	12	25	28	30	6.6	0.46	0.92	0.74(0.61-0.85)

Table S3. Operative performance of stroke volume variation of fluid responsiveness from included studies. Values are expressed as pooled values (95% confidence interval) or median (IQR). AUC, area under curve reported by each study, NR, not reported.

Study	Year	Number total of patients	True positive	Fluid responders	True negative	Non-fluid responders	Cut-off	Sensitivity	Specificity	AUC
Lafanechere et al (7)	2006	22	9	10	10	12	8	0.9	0.83	0.95±0.04
Monnet et al (8)	2006	71	36	37	32	34	10	0.97	0.94	0.91±0.06
Monnet et al (8)	2006	71	22	37	27	34	12	0.6	0.85	0.75±0.06
Maizel et al (13)	2007	34	11	17	15	17	12	0.63	0.89	0.89±0.06
Maizel et al (13)	2007	34	12	17	15	17	12	0.69	0.89	0.90±0.06
Lamia et al (14)	2007	24	10	13	11	11	12.5	0.77	1	NR
Lamia et al (14)	2007	24	16	16	8	8	8	1	1	NR
Monnet et al (27)	2009	34	21	23	11	11	10	0.91	1	0.93(0.79-0.99)
Monnet et al (27)	2009	34	11	23	10	11	11	0.48	0.91	0.67(0.49-0.80)
Biais et al (25)	2009	30	20	20	8	10	13	1	0.8	0.96±0.03
Biais et al (25)	2009	30	17	20	9	10	16	0.85	0.9	0.92±0.05
Thiel et at (26)	2009	102	38	47	51	55	15	0.81	0.93	0.89±0.04
Préau et al (31)	2010	34	12	14	18	20	10	0.86	0.9	0.94±0.04
Préau et al (31)	2010	34	11	14	17	20	9	0.79	0.85	0.86±0.08
Préau et al (31)	2010	34	12	14	16	20	8	0.86	0.8	0.93±0.04
Huang et al (36)	2011	30	13	15	14	15	11	0.86	0.93	0.94±0.03
Dong et al (52)	2012	32	15	22	8	10	8.8	0.72	0.8	0.86±0.06
Dong et al (52)	2012	32	16	22	8	10	12.7	0.72	0.8	0.80±0.07
Monge et al (53)	2012	37	20	21	15	16	12	0.95	0.93	0.97±0.03
Monge et al (53)	2012	37	19	21	15	16	5	0.9	0.93	0.94±0.03
Monge et al (53)	2012	37	14	21	13	16	11	0.66	81.2	0.73±0.09
Fellahi et al (41)	2012	25	7	14	10	11	6	0.5	0.91	0.72(0.50-0.88)
Fellahi et al (42)	2012	25	13	14	8	11	3	0.93	0.73	0.81(0.62-0.94)
Monnet et al (45)	2012	39	17	17	20	22	11	1	0.91	0.95(0.88-1.03)
Monnet et al (44)	2012	26	14	15	10	11	10	0.93	0.91	0.91±0.06

Study	Year	Number total of patients	True positive	Fluid responders	True negative	Non-fluid responders	Cut-off	Sensitivity	Specificity	AUC
Monnet et al (44)	2012	28	14	15	13	13	10	0.94	1	0.94±0.05
Monnet et al (56)	2013	40	20	21	18	19	10	0.95	0.95	0.98(0.88-1.00)
Monnet et al (56)	2013	40	15	21	19	19	5	0.71	1	0.93(0.81-0.99)
Saugel et al (66)	2013	24	5	7	4	17	15	0.71	0.23	NR
Saugel et al (66)	2013	24	3	7	14	17	15	0.42	0.82	NR
Saugel et al (66)	2013	24	7	7	3	17	15	1	0.17	NR
Kupersztych-Hagege et al (67)	2013	48	16	19	28	29	9	0.84	0.97	0.87±0.06
Ibarra-Estrada et al (82)	2015	59	19	30	14	19	15	0.63	0.72	0.69(0.56-0.80)
Airapetian et al (84)	2015	59	9	29	29	30	42	0.31	0.97	0.62±0.07
Airapetian et al (84)	2015	59	27	29	10	30	2.1	0.93	0.33	0.62±0.07
Airapetian et al (84)	2015	59	15	29	26	30	10	0.52	0.87	0.78±0.06
Fisher et al (91)	2016	78	42	55	13	23	3	0.76	0.57	0.67(0.55-0.77)
Kim et al (92)	2016	43	11	15	22	28	7.3	0.71	0.79	0.77(0.61-0.92)
Sobczyk et al (94)	2016	35	19	24	9	11	15	0.79	0.81	0.8
Kridge et al (98)	2016	33	8	10	21	23	9	0.8	0.91	0.85(0.63-1.00)
Renner et al (109)	2017	46	17	21	18	25	11	0.81	0.72	0.86(0.75-0.96)
Renner et al (109)	2017	46	16	21	18	25	8	0.76	0.72	0.78(0.65-0.92)
El Hadouti et al (107)	2017	41	21	22	10	19	5	0.95	0.52	NR
El Hadouti et al (107)	2017	41	22	22	9	19	5	1	0.47	NR
El Hadouti et al (107)	2017	41	19	22	12	19	8	0.86	0.63	NR
El Hadouti et al (107)	2017	41	21	22	12	19	8	0.95	0.63	NR
El Hadouti et al (107)	2017	41	18	22	16	19	10	0.81	0.84	NR
El Hadouti et al (107)	2017	41	17	22	16	19	10	0.77	0.84	NR

Study	Year	Number total of patients	True positive	Fluid responders	True negative	Non-fluid responders	Cut-off	Sensitivity	Specificity	AUC
El Hadouti et al (107)	2017	41	18	22	17	19	11	0.81	0.89	NR
El Hadouti et al (107)	2017	41	15	22	19	19	11	0.68	1	NR
El Hadouti et al (107)	2017	41	15	22	17	19	12	0.68	0.89	NR
El Hadouti et al (107)	2017	41	14	22	19	19	12	0.63	1	NR
Ma et al (111)	2017	70	35	35	29	35	12.84	1	0.82	0.91(0.82-0.97)
Wu et al (112)	2017	62	25	28	30	34	12.45	0.89	0.88	0.92
Wu et al (112)	2017	62	23	28	30	34	10.6	0.82	0.88	0.89
Xu et al (110)	2017	34	14	14	19	20	10	1	0.95	0.96(0.84-0.99)
Xu et al (110)	2017	34	13	14	15	20	10	0.93	0.75	0.86(0.70-0.95)
Si et al (113)	2018	40	24	26	12	14	12	0.92	0.86	0.94±0.03
Si et al (113)	2018	40	21	26	12	14	7	0.81	0.86	0.85±0.06
Si et al (113)	2018	40	18	26	11	14	5	0.69	0.78	0.77±0.07
Si et al (113)	2018	40	16	26	10	14	7	0.69	0.72	0.76±0.07
Si et al (113)	2018	40	16	26	11	14	5	0.62	0.79	0.62±0.09
Trifi et al (120)	2019	30	18	19	8	11	15	0.94	0.72	0.84±0.04
Beurton et al (121)	2019	30	14	15	15	15	10	0.93	1	0.98±0.02
Hou et al (131)	2020	102	33	53	37	49	17	0.62	0.75	0.73(0.63-0.81)
Hou et al (131)	2020	102	31	53	43	49	21	0.58	0.87	0.75(0.65-0.83)
Hou et al (131)	2020	102	42	53	24	49	4	0.79	0.48	0.65(0.55-0.74)
Beurton et al (125)	2019	72	31	34	30	38	9	0.91	0.79	0.89(0.80-0.95)
Hou et al (131)	2019	102	44	53	26	49	7	0.83	0.53	0.70(0.60-0.79)
Bataille et al (137)	2021	100	30	50	42	50	10	0.61	0.85	0.77(0.64-0.91)
Taccheri et al (142)	2021	30	15	15	14	15	<20%	1	0.93	0.98±0.02
Taccheri et al (142)	2021	30	14	15	14	15	< - 2	0.93	0.93	0.98±0.03

Study	Year	Number total of patients	True positive	Fluid responders	True negative	Non-fluid responders	Cut-off	Sensitivity	Specificity	AUC
Taccheri et al (142)	2021	30	12	15	15	15	<19%	0.8	1	0.98±0.04
Taccheri et al (142)	2021	30	11	15	15	15	<-2	0.73	1	0.98±0.05
Hamzaoui et al (134)	2021	54	19	22	22	32	-1	0.87	0.68	0.78(0.65-0.88)
Taccheri et al (142)	2021	30	15	15	10	15	> 4%	1	0.67	0.98±0.06
Taccheri et al (142)	2021	30	15	15	11	15	> 1	1	0.6	0.98±0.07
Giraud et al (135)	2021	33	14	15	17	18	4	0.93	0.94	0.92±0.05
Elsayed et al (143)	2021	46	14	16	24	30	2.5	0.87	0.8	0.87(0.77-0.98)
Elsayed et al (143)	2021	46	15	16	26	30	6.5	0.93	0.86	0.94(0.88-1.00)
Shi et al (145)	2022	35	10	14	16	21	2	0.71	0.76	0.76(0.59-0.89)
Mallat et al (148)	2022	270	148	164	94	106	-18.2	0.9	0.88	0.92(0.88-0.95)
Mallat et al (148)	2022	270	147	164	93	106	-2	0.89	0.87	0.92(0.88-0.95)

Table S4. Operative performance of passive leg raising of fluid responsiveness from included studies. Values are expressed as pooled values (95% confidence interval) or median (IQR). AUC. area under curve reported by each study; NR, not reported.

Study	Year	Number total of patients	True positive	Fluid responders	True negative	Non-fluid responders	Cut-off	Sensitivity	Specificity	AUC
Monnet et al (27)	2009	34	20	23	11	11	**5**	0.87	1	0.95(0.82-0.99)
Monnet et al (27)	2009	34	21	23	11	11	**5**	0.91	1	0.97(0.84-0.99)
Monnet et al (27)	2009	34	15	23	9	11	**4**	0.67	0.82	0.71(0.52-0.85)
Monnet et al (45)	2012	39	17	17	20	22	**5**	1	0.91	0.97(0.91-1.03)
Monnet et al (44)	2012	26	14	15	10	11	**5**	0.93	0.91	0.97±0.03
Monnet et al (44)	2012	28	14	15	12	13	**5**	0.93	0.92	0.93±0.05
Silva et al(68)	2013	34	9	10	21	24	**5**	0.9	0.88	0.90(0.75-0.98)
Silva et al(68)	2013	34	13	13	19	21	**6**	1	0.9	0.96(0.82-0.99)
Guinot et al (78)	2014	42	23	28	10	14	**13**	0.82	0.71	0.78(0.63-0.89)
Guinot et al (78)	2014	42	23	28	8	14	**13**	0.82	0.57	0.68(0.51-0.81)
Myatra et al (99)	2017	30	14	16	13	14	**4.1**	0.88	0.93	0.95(0.88-1.00)
Yonis et al (100)	2017	33	5	15	19	19	**10**	0.33	1	0.65(0.46-0.84)
Jozwiak et al (108)	2017	30	14	15	15	15	**4**	0.93	1	0.98(0.85-1.00)
Jozwiak et al (108)	2017	30	14	15	15	15	**5**	0.93	1	0.93(0.78-0.99)
Blais et al (103)	2017	41	20	20	17	21	**5**	1	0.81	0.91(0.81-1.00)
Blais et al (103)	2017	41	11	20	15	21	**1**	0.55	0.71	0.44-0.80)
Georges et al (114)	2018	50	25	28	21	22	**9**	0.89	0.95	0.96±0.03
Georges et al (114)	2018	50	18	28	17	22	**8.5**	0.64	0.77	0.70±0.07
Depret el at (122)	2019	28	12	14	13	14	**3**	0.86	0.93	0.95(0.79-0.99)
Depret el at (122)	2019	28	14	14	14	14	**4**	1	1	1(0.88-1.00)
Depret el at (122)	2019	28	10	14	12	14	**3**	0.71	0.86	0.80(0.61-0.93)
Depret el at (122)	2019	28	10	14	12	14	**2**	0.71	0.86	0.75(0.55-0.89)
Messina et al (123)	2019	40	19	21	16	19	**3.6**	0.89	0.85	0.93(0.84-1.00)

Study	Year	Number total of patients	True positive	Fluid responders	True negative	Non-fluid responders	Cut-off	Sensitivity	Specificity	AUC
Messina et al (123)	2019	40	19	21	16	19	4.7	0.89	0.85	0.95(0.88-1.00)
Xu et al (119)	2019	75	29	36	36	39	5	0.8	0.92	0.90(0.83-0.97)
Xu et al (119)	2019	75	11	36	37	39	3	0.31	0.94	0.75(0.63-0.86)
Xu et al (119)	2019	75	22	36	29	39	5	0.6	0.74	0.71(0.59-0.83)
Weil et al (132)	2020	65	15	25	27	40	10	0.6	0.67	0.70(0.57-0.81)
Weil et al (132)	2020	50	13	19	25	31	14	0.68	0.8	0.78(0.64-0.89)
Lizuka et al (138)	2021	41	10	16	17	25	1.02	0.64	0.68	0.56(0.37-0.74)
Lizuka et al (138)	2021	41	10	16	20	25	1.04	0.6	0.81	0.68(0.52-0.85)
Lizuka et al (138)	2021	41	10	16	17	25	1	0.6	0.68	0.61(0.42-0.80)
Lizuka et al (138)	2021	41	8	16	14	25	1	0.52	0.56	0.42(0.24-0.60)
Messina et al (139)	2021	102	46	53	22	49	3.4	0.85	0.45	0.67(0.57-0.76)
Messina et al (139)	2021	103	49	53	23	50	3.4	0.92	0.45	0.73(0.63-0.81)
Shi et al (144)	2022	84	37	42	39	42	3.2	0.88	0.93	0.93(0.87-0.98)

Table S5. Operative performance of end-expiratory occlusion test of fluid responsiveness from included studies. Values are expressed as pooled values (95% confidence interval) or median (IQR). AUC, area under curve reported by each study, NR, not reported.

Study	Year	Number total of patients	True positive	Fluid responders	True negative	Non-fluid responders	Cut-off	Sensitivity	Specificity	AUC
Muller et al (40)	2011	39	20	21	14	18	10	0.95	0.78	NR
Wu et al (79)	2014	50	20	27	22	23	9	0.74	0.95	0.91±0.04
Wu et al (79)	2014	50	25	27	21	23	6	0.93	0.91	0.95±0.03
Wu et al (79)	2014	50	27	27	23	23	10	1	1	0.96±0.03
Guinot et al (85)	2015	73	24	27	41	46	7	0.89	0.89	0.93(0.84-0.97)
Mallat et al (86)	2015	49	17	22	20	27	5.2	0.77	0.74	0.78(0.68-0.88)
Mallat et al (86)	2015	49	19	22	24	27	-2	0.86	0.89	0.91(0.80-0.97)
Mallat et al (86)	2015	49	19	22	23	27	-2	0.86	0.85	0.92(0.81-0.98)
Lagreze et al (97)	2016	40	5	15	25	25	3	0.33	1	0.74(0.67-0.97)
Biais et al (104)	2017	88	25	28	40	60	2	0.89	0.67	0.83(0.75-0.92)
Biais et al (104)	2017	88	26	28	51	60	6	0.93	0.85	0.95(0.90-0.99)
Ali et al (118)	2019	33	13	15	18	18	5.8	0.87	1	0.96(0.83-0.99)
Fot et al (124)	2019	33	11	14	12	18	2	0.8	0.67	0.75
Fot et al (124)	2019	33	12	14	12	18	2	0.87	0.67	0.77
Lee et al (127)	2020	50	26	30	17	20	5	0.87	0.85	0.90(0.82-0.99)
Messina et al (139)	2021	103	52	53	43	50	4	0.98	0.86	0.95(0.88-0.98)

Table S6. Operative performance of mini-fluid challenge of fluid responsiveness from included studies. Values are expressed as pooled values (95% confidence interval) or median (IQR). AUC, area under curve reported by each study; NR, not reported.

Study	Year	Number total of patients	True positive	Fluid responders	True negative	Non-fluid responders	Cut-off	Sensitivity	Specificity	AUC
Myatra et al (99)	2017	30	15	16	14	14	3.5	0.94	1	0.99(0.98-1.00)
Myatra et al (99)	2017	30	14	16	14	14	2.5	0.88	1	0.97(0.92-1.00)
Myatra et al (99)	2017	30	15	16	14	14	48	0.94	1	0.97(0.92-1.00)
Myatra et al (99)	2017	30	14	16	13	14	43	0.88	0.93	0.96(0.89-1.00)
Yonis et al (100)	2017	33	15	15	8	18	29	1	0.4	0.59(0.31-0.88)
Messina et al (123)	2019	40	18	19	16	21	13.3	0.94	0.76	0.94(0.82-0.99)
Messina et al (123)	2019	40	15	19	20	21	12.1	0.78	0.95	0.93(0.80-0.98)
Jun et al (126)	2019	38	22	24	12	14	1	0.92	0.86	0.95(0.83-0.99)
Jun et al (126)	2019	38	11	24	14	14	1	0.46	1	0.76(0.60-0.89)
Jun et al (126)	2019	38	20	24	11	14	1	0.83	0.79	0.87(0.72-0.96)
Jun et al (126)	2019	38	16	24	11	14	1	0.67	0.79	0.71(0.55-0.85)
Messina et al (133)	2020	40	18	19	20	21	3.50%	0.95	0.94	0.96(0.87-1.03)
Messina et al (133)	2020	40	18	19	20	21	12.2	0.95	0.94	0.96(0.89-1.02)
Hamzaoui et al (134)	2021	54	15	22	24	32	8	0.69	0.76	0.73 (0.60-0.84)
Taccheri et al (142)	2021	30	14	15	13	15	2	1	0.93	0.94±0.04
Taccheri et al (142)	2021	30	14	15	15	15	20%	0.93	0.93	0.98±0.03
Taccheri et al (142)	2021	30	15	15	10	15	1	1	0.67	0.82±0.08
Taccheri et al (142)	2021	30	14	15	11	15	20%	0.93	0.73	0.94±0.04
Taccheri et al (142)	2021	30	15	15	10	15	1	1	0.67	0.88±0.06
Taccheri et al (142)	2021	30	15	15	9	15	4%	1	0.6	0.92±0.05

Study	Year	Number total of patients	True positive	Fluid responders	True negative	Non-fluid responders	Cut-off	Sensitivity	Specificity	AUC
Elsayed et al (143)	2021	46	15	16	28	30	1	0.93	0.93	0.95(0.89-1.00)
Shi et al (144)	2022	84	41	42	36	42	3.5	0.98	0.86	0.94±0.03
Xu et al (147)	2022	76	26	31	38	45	2	0.8	0.8	0.90(0.80-0.95)
Botros et al (149)	2023	48	19	20	19	28	2.5	0.95	0.68	0.86(0.76-0.96)
Botros et al (149)	2023	48	16	20	21	28	29%	0.82	0.75	0.83(0.72-0.94)

Table S7. Operative performance of tidal-volume challenge of fluid responsiveness from included studies. Values are expressed as pooled values (95% confidence interval) or median (IQR). AUC, area under curve reported by each study.

Study	Year	Risk of bias/Patient selection	Risk of bias/Index test	Risk of bias/Reference Standard	Risk of bias/Flow and timing	Applicability/Patient selection	Applicability/Index test	Applicability/Reference Standard
Michard et al (1)	2000	Unclear	Low	Low	Low	Low	Low	Low
Berkenstadt et al (2)	2001	Unclear	Low	Low	Low	Low	Low	Low
Kramer et al (3)	2004	Low	Low	Low	Low	Low	Low	Low
De Backer et al (4)	2005	Low	Low	Low	Low	Low	Low	Low
Feissel et al (5)	2005	Low	Low	Low	Low	Low	Low	Low
Hofer et al (6)	2005	Low	Low	Low	Low	Low	Low	Low
Lafanechere et al (7)	2006	Low	Low	Low	Low	Low	Low	Low
Monnet et al (8)	2006	Low	Low	Low	Low	Low	Low	Low
Charron et al (9)	2006	Low	Low	Low	Low	Low	Low	Low
Cannesson et al (10)	2007	Low	Low	Low	Low	Low	Low	Low
Feissel et al (11)	2007	Low	Low	Low	Low	Low	Low	Low
Wyffels et al (12)	2007	Low	Low	Low	Low	Low	Low	Low
Maizel et al (13)	2007	Low	Low	Low	Low	Low	Low	Low
Lamia et al (14)	2007	Low	Low	Low	Low	Low	Low	Low
Auler et al (15)	2008	Unclear	Low	Low	Low	Low	Low	Low
Cannesson et al (16)	2008	Low	Low	Low	Low	Low	Low	Low
Huang et al (17)	2008	Low	Low	Low	Low	Low	Low	Low
Derichard et al (18)	2009	Low	Low	Low	Low	Low	Low	Low
de Wall et al (19)	2009	Low	Low	Low	Low	Low	Low	Low

Study	Year	Risk of bias/Patient selection	Risk of bias/Index test	Risk of bias/Reference Standard	Risk of bias/Flow and timing	Applicability/Patient selection	Applicability/Index test	Applicability/Reference Standard
Cannesson et al (20)	2009	Low	Low	Low	Low	Low	Low	Low
Monge et al (21)	2009	Low	Low	Low	Low	Low	Low	Low
Vistisen et al (22)	2009	Low	Low	Low	Low	Low	Low	Low
Vallee et al (23)	2009	Low	Low	Low	Low	Low	Low	Low
Lahner et al (24)	2009	Low	Low	Low	Low	Low	Low	Low
Biais et al (25)	2009	Unclear	Low	Low	Low	Low	Low	Low
Thiel et at (26)	2009	Low	Low	Low	Low	Low	Low	Low
Monnet et al (27)	2009	Low	Low	Low	Low	Low	Low	Low
Muller et al (28)	2010	Unclear	Low	Low	Low	Low	Low	Low
Biais et al (29)	2010	Low	Low	Low	Low	Low	Low	Low
Zimmermann et al (30)	2010	Unclear	Low	Low	Low	Low	Low	Low
Préau et al (31)	2010	Low	Low	Low	Low	Low	Low	Low
Lakhal et al (32)	2011	Low	Low	Low	Low	Low	Low	Low
Geerts et al (33)	2011	Unclear	Low	Low	Low	Low	Low	Low
Desgranges et al (34)	2011	Unclear	Low	Low	Low	Low	Low	Low
Machare-Delgado et al (35)	2011	Low	Low	Low	Low	Low	Low	Low
Huang et al (36)	2011	Low	Low	Low	Low	Low	Low	Low
Broch et al (37)	2011	Low	Low	Low	Low	Low	Low	Low
Geerts et al (38)	2011	Unclear	Low	Low	Low	Low	Low	Low
Loupec et al (39)	2011	Low	Low	Low	Low	Low	Low	Low
Muller et al (40)	2011	Low	Low	Low	Low	Low	Low	Low
Fellahi et al (41)	2012	Low	Low	Low	Low	Low	Low	Low

Study	Year	Risk of bias/Patient selection	Risk of bias/Index test	Risk of bias/Reference Standard	Risk of bias/Flow and timing	Applicability/Patient selection	Applicability/Index test	Applicability/Reference Standard
Fellahi et al (42)	2012	Low	Low	Low	Low	Low	Low	Low
Khwannimit et al (43)	2012	Low	Low	Low	Low	Low	Low	Low
Monnet et al (44)	2012	Low	Low	Low	Low	Low	Low	
Monnet et al (45)	2012	Low	Low	Low	Low	Low	Low	Low
Cecconi et al (46)	2012	Low	Low	Low	Low	Low	Low	Low
Yazigi et al (47)	2012	Low	Low	Low	Low	Low	Low	Low
Oliveira-Costa et al (48)	2012	Low	Low	Low	Low	Low	Low	Low
Biais et al (49)	2012	Low	Low	Low	Low	Low	Low	Low
Fu et al (50)	2012	Low	Low	Low	Low	Low	Low	Low
J Li et al (51)	2012	Low	Low	Low	Low	Low	Low	Low
Dong et al (52)	2012	Low	Low	Low	Low	Low	Low	Low
Monge et al (53)	2012	Low	Low	Low	Low	Low	Low	Low
Broch et al (54)	2012	High	Low	Low	Low	Low	Low	Low
Monnet et al (55)	2013	Low	Low	Low	Low	Low	Low	Low
Monnet et al (56)	2013	Low	Low	Low	Low	Low	Low	Low
Davies et al (57)	2013	Low	Low	Low	Low	Low	Low	Low
Nordstrom et al (58)	2013	Low	Low	Low	Low	Low	Low	Low
Fischer et al (59)	2013	Low	Low	Low	Low	Low	Low	Low
Ishihara et al (60)	2013	Low	Low	Low	Low	Low	Low	Low
Drvar et al (61)	2013	Low	Low	Low	Low	Low	Low	Low
Freitas et al (62)	2013	Low	Low	Low	Low	Low	Low	Low
Trepte et al (63)	2013	Low	Low	Low	Low	Low	Low	Low
Yang et al (64)	2013	Low	Low	Low	Low	Low	Low	Low

Study	Year	Risk of bias/Patient selection	Risk of bias/Index test	Risk of bias/Reference Standard	Risk of bias/Flow and timing	Applicability/Patient selection	Applicability/Index test	Applicability/Reference Standard
Kim et al (65)	2013	Low	Low	Low	Low	Low	Low	Low
Saugel et al (66)	2013	Low	Low	Low	Low	Low	Low	Low
Kupersztych-Hagege et al (67)	2013	Low	Low	Low	Low	Low	Low	Low
Silva et al (68)	2013	Low	Low	Low	Low	Low	Low	Low
Pei et al (69)	2014	Low	Low	Low	Low	Low	Low	Low
Hoiseth et al (70)	2014	Unclear	Low	Low	Low	Low	Low	Low
Guarracino et al (71)	2014	Low	Low	Low	Low	Low	Low	Low
Song et al (72)	2014	Unclear	Low	Low	Low	Low	Low	Low
Shim et al (73)	2014	Low	Low	Low	Low	Low	Low	Low
Fisher et al (74)	2014	Low	Low	Low	Low	Low	Low	Low
Kurts et al (75)	2014	Low	Low	Low	Low	Low	Low	Low
Guinot et al (76)	2014	Low	Low	Low	Low	Low	Low	Low
Kang et at (77)	2014	Low	Low	Low	Low	Low	Low	Low
Guinot et al (78)	2014	Low	Low	Low	Low	Low	Low	Low
Wu et al (79)	2014	Low	Low	Low	Low	Low	Low	Low
Messina et al (80)	2015	Low	Low	Low	Low	Low	Low	Low
Zhao et al (81)	2015	Low	Low	Low	Low	Low	Low	Low
Ibarra-Estrada et al (82)	2015	Low	Low	Low	Low	Low	Low	Low
Angappan et al (83)	2015	Low	Low	Low	Low	Low	Low	Low
Airapetian et al (84)	2015	Low	Low	Low	Low	Low	Low	Low
Guinot et al (85)	2015	Low	Low	Low	Low	Low	Low	Low
Mallat et al (86)	2015	Low	Low	Low	Low	Low	Low	Low

Study	Year	Risk of bias/Patient selection	Risk of bias/Index test	Risk of bias/Reference Standard	Risk of bias/Flow and timing	Applicability/Patient selection	Applicability/Index test	Applicability/Reference Standard
Theerawit et aL (87)	2016	Low	Low	Low	Low	Low	Low	Low
Lee et al (88)	2016	Low	Low	Low	Low	Low	Low	Low
Cherpanath et al (89)	2016	Unclear	Low	Low	Low	Low	Low	Low
Zhang et al (90)	2016	Unclear	Low	Low	Low	Low	Low	Low
Fisher et al (91)	2016	Low	Low	Low	Low	Low	Low	Low
Kim et al (92)	2016	Low	Low	Low	Low	Low	Low	Low
de Oliveira et al (93)	2016	Low	Low	Low	Low	Low	Low	Low
Sobczyk et al (94)	2016	Low	Low	Low	Low	Low	Low	Low
Liu et al (95)	2016	Low	Low	Low	Low	Low	Low	Low
Vistisen et al (96)	2016	Unclear	Low	Low	Low	Low	Low	Low
Lagreze et al (97)	2017	Low	Low	Low	Low	Low	Low	Low
Kridge et al (98)	2016	Low	Low	Low	Low	Low	Low	Low
Myatra et al (99)	2017	Low	Low	Low	Low	Low	Low	Low
Yonis et al (100)	2017	Low	Low	Low	Low	Low	Low	Low
Renner et al (101)	2017	Unclear	Low	Low	Low	Low	Low	Low
Min et al (102)	2017	Low	Low	Low	Low	Low	Low	Low
BIais et al (103)	2017	High	Low	Low	Low	Low	Low	Low
Biais et al (104)	2017	Low	Low	Low	Low	Low	Low	Low
Messina et al (105)	2017	Low	Low	Low	Low	Low	Low	Low
Lu et al (106)	2017	Low	Low	Low	Low	Low	Low	Low
El Hadouti et al (107)	2017	Low	Low	Low	Low	Low	Low	Low

Study	Year	Risk of bias/Patient selection	Risk of bias/Index test	Risk of bias/Reference Standard	Risk of bias/Flow and timing	Applicability/Patient selection	Applicability/Index test	Applicability/Reference Standard
Jozwiak et al (108)	2017	Low	Low	Low	Low	Low	Low	Low
Renner et al (109)	2017	Unclear	Low	Low	Low	Low	Low	Low
Xu et al (110)	2017	Low	Low	Low	Low	Low	Low	Low
Guo-Guang Ma et al (111)	2018	Low	Low	Low	Low	Low	Low	Low
Wu et al (112)	2018	Low	Low	Low	Low	Low	Low	Low
Si et al (113)	2018	Low	Low	Low	Low	Low	Low	Low
Georges et al (114)	2018	High	Low	Low	Low	Low	Low	Low
Joosten et al (115)	2019	Low	Low	Low	Low	Low	Low	Low
Ali et al (116)	2019	Low	Low	Low	Low	Low	Low	Low
He et al (117)	2019	Low	Low	Low	Low	Low	Low	Low
Ali et al (118)	2019	Low	Low	Low	Low	Low	Low	Low
Xu et al (119)	2019	Low	Low	Low	Low	Low	Low	Low
Trifi et al (120)	2019	Low	Low	Low	Low	Low	Low	Low
Beurton et al (121)	2019	Low	Unclear	Low	Low	Low	Low	Low
Depret el at (122)	2019	Low	Low	Low	Low	Low	Low	Low
Messina et al (123)	2019	Low	Low	Low	Low	Low	Low	Low
Fot et al (124)	2019	Low	Low	Low	Low	Low	Low	Low
Beurton et al (125)	2019	Low	Low	Low	Low	Low	Low	Low
Jun et al (126)	2019	Low	Low	Low	Low	Low	Low	Low
Lee et al (127)	2020	Low	Low	Low	Low	Low	Low	Low

Study	Year	Risk of bias/Patient selection	Risk of bias/Index test	Risk of bias/Reference Standard	Risk of bias/Flow and timing	Applicability/Patient selection	Applicability/Index test	Applicability/Reference Standard
Wang et al (128)	2020	Low	Low	Low	Low	Low	Low	Low
Bapteste et al (129)	2020	Low	Low	Low	Low	Low	Low	Low
Bubenek et al (130)	2020	Low	Low	Low	Low	Low	Low	Low
Hou et al (131)	2020	Low	Low	Low	Low	Low	Low	Low
Weil et al (132)	2020	Low	Low	Low	Low	Low	Low	Low
Messina et al (133)	2020	Low	Low	Low	Low	Low	Low	Low
Hamzaoui et al (134)	2020	Low	Low	Low	Low	Low	Low	Low
Giraud et al (135)	2020	Low	Low	Low	Low	Low	Low	Low
Kaur et al (136)	2021	Low	Low	Low	Low	Low	Low	Low
Bataille et al (137)	2021	Low	Low	Low	Low	Low	Low	Low
Lizuka et al (138)	2021	Low	Low	Low	Low	Low	Low	Low
Messina et al (139)	2021	Low	Low	Low	Low	Low	Low	Low
Watanabe et al (140)	2021	Low	Low	Low	Low	Low	Low	Low
Chen et al (141)	2021	Low	Low	Low	Low	Low	Low	Low
Taccheri et al (142)	2021	Low	Low	Low	Low	Low	Low	Low
Elsayed et al (143)	2021	Low	Low	Low	Low	Low	Low	Low
Shi et al (144)	2022	Low	Low	Low	Low	Low	Low	Low
Shi et al (145)	2022	Low	Low	Low	Low	Low	Low	Low
Abdullah et al (146)	2022	Low	Low	Low	Low	Low	Low	Low
Xu et al (147)	2022	Low	Low	Low	Low	Low	Low	Low

Study	Year	Risk of bias/Patient selection	Risk of bias/Index test	Risk of bias/Reference Standard	Risk of bias/Flow and timing	Applicability/Patient selection	Applicability/Index test	Applicability/Reference Standard
Mallat et al (148)	2022	Low	Low	Low	Low	Low	Low	Low
Botros et al (149)	2023	Low	Low	Low	Low	Low	Low	Low

Table S8. Risk of bias of the trials as assessed by QUADAS-2 criteria.

Predictor of fluid responsiveness evaluated	Intercept (SD)	P value by Egger test
PPV	0.11 (0.27)	<0.01
SVV	0.53(0.36)	<0.01
PLR	1.34(0.33)	<0.01
EEOT	-1.15(0.42)	<0.01
MFC	0.92(1.10)	0.10
VTC	1.12(0.22)	<0.01

Table S9. Asymmetry assessment by Egger test. EEOT, end-expiratory occlusion test, PPV, pulse pressure variation; SVV, stroke volume variation; PLR, passive leg raising; EEOT, end-expiratory occlusion test; MFC, mini-fluid challenge; VTC, tidal volume challenge. Values are expressed as pooled data (95% confidence interval).

Predictor of Fluid Responsiveness	DOR	95% Confidence Interval	I^2
PPV	7.5	4.8-24.5	66.3
SVV	6.9	5.0-9.4	66.5
PLR	11.3	8.3-15.3	67.4
EEOT	6.7	4.0-11.3	77.1
VTC	19.4	11.6-33.1	47.0

Table S10. Asymmetry fitted by the trim-and-fill method for predictors of fluid responsiveness. EEOT, end-expiratory occlusion test, I^2, inconsistency; PPV, pulse pressure variation; SVV, stroke volume variation; PLR, passive leg raising; EEOT, end-expiratory occlusion test. Values are expressed as pooled data (95% confidence interval).

Subgroup	Predictor evaluated	Number Of Studies	Unadjusted DOR (95%CI)	Adjusted DOR (95% CI)	P value by subgroup analysis	P value (by meta-regression)	I2(%)
Tidal volume > 8 ml/kg<br>Yes<br>No	PPV	39<br>64	16.6 (12.9 – 21.7)	24.0(17.1-33.5)<br>10.2(7.0-14.8)	<0.01	<0.01<br><0.01	50.8
Tidal volume	PPV	103	16.6 (12.9 – 21.7)	DOR = 1.2 (ml/kg IBW Vt)	NA	<0.01	50.8
Tidal volume > 8 ml/kg<br>Yes<br>No	SVV	51<br>18	14.4(10.7-19.4)	14.8(10.5-21.0)<br>13.5(7.6-24.0)	0.78	NA	54.4
Lung compliance	PPV	38	16.6 (12.9 – 21.7)	DOR = 1.05(L/cmH20 Lung Compliance)	<0.01	<0.01	50.9
Thermodilution<br>Yes<br>No	PPV	54<br>49	16.6 (12.9 – 21.7)	14.8(10-3-21.2)<br>19.1(13.1-27.8)	0.39	NA	54.2
Thermodilution<br>Yes<br>No	SVV	22<br>47	14.4(10.7-19.4)	11.2(6.7-19.1)<br>16.2(11.3-23.1)	0.27	NA	53.7
Thermodilution<br>Yes<br>No	PLR	22<br>59	22.6(17.0-30.2)	14.2(8.1-24.7)<br>24.2(17.3-33.9)	0.1	NA	47.5
Thermodilution<br>Yes<br>No	EEOT	16<br>20	17.8(10.7-30.0)	67.8(30.0-153.4)<br>9.4(5.6-15.7)	<0.01	<0.01<br><0.01	51.3
Thermodilution<br>Yes<br>No	MFC	5<br>11	37.3(21.8-64.0)	16.4(7.7-34.8)<br>57.0(31.5-102.7)	0.01	0.01<br><0.01	9.0
Thermodilution<br>Yes<br>No	VTC	6<br>19	46.6(26.7-81.3)	71.0(21.0-239.9)<br>41.9(22.2-78.9)	0.45	NA	28.5
Threshold >10%<br>Yes<br>No	PPV	82<br>21	16.6 (12.9 – 21.7)	19.8(14.7-26.6)<br>9.6(5.8-16.9)	<0.01	<0.01<br><0.01	51.7

Threshold >10%<br>Yes<br>No	SVV	 43<br>26	 14.4(10.7-19.4)	 15.7(10.6-23.2)<br>13.0(8.1-20.4)	 0.52	 NA	 54.4
Threshold >10%<br>Yes<br>No	PLR	 68<br>15	 22.6(17.0-30.2)	 21.6(15.8-29.7)<br>29.3(13.9-61.9)	 0.47	 NA	 53.6
Threshold >10%<br>Yes<br>No	 EEOT	 23<br>13	 17.8(10.7-30.0)	 25.7(13.0-50.5)<br>12.3(5.5-28.0)	 0.17	 NA	 64.6
Threshold >10%<br>Yes<br>No	MFC	 13<br>3	 37.3(21.8-64.0)	 34.0(18.0-63.6)<br>55.0(16.5-183.0)	 0.48	 NA	 34.3
Driving pressure	EEOT	23	17.8(10.7-30.0)	DOR =1.1 (cmH20)	0.26	NA	67.9
Tidal volume	EEOT	38	17.8(10.7-30.0)	DOR = 12.27(Vt ml/kg)	0.39	NA	72.8
Prone position<br>Yes<br>No	 VTC	 4<br>21	 46.6(26.7-81.3)	 70.0(17.2-283.43)<br>43.5(23.5-80.7)	 0.54	 NA	 35.4
The MFC was included in Fluid responsiveness assessment.<br>Yes<br>No	MFC	 13<br>3	37.3(21.8-64.0)	 45.1(24.4-83.1)<br>18.9(5.8-61.0)	 0.19	 NA	 28.4

Table S11. Adjusted operative performance of the technical variables for predictors of fluid responsiveness evaluated. DOR: diagnostic odd ratio; I^2: inconsistency. NA: not applicable; PPV, pulse pressure variation, PLR passive leg raising, EEOT, end-expiratory occlusion test; MFC, mini-fluid challenge; VTC, tidal-volume challenge; ABF, aortic blood flow; CO, cardiac output; CI, cardiac index; SVI, stroke volume index; SV, stroke volume; VTI, velocity-time integral. Values are expressed as pooled data (95% confidence interval).

Question: Should Pulse Pressure Variation be used to diagnose an increase in cardiac output in critical care patients after a fluid challenge?

Sensitivity	0.77 (95% CI: 0.74 to 0.80)
Specificity	0.79 (95% CI: 0.76 to 0.82)

Prevalence	40%	50%	60%

Outcome	№ of studies (№ of patients)	Study design	Factors that may decrease certainty of evidence					Effect per 1,000 patients tested			Test accuracy CoE
			Risk of bias	Indirectness	Inconsistency	Imprecision	Publication bias	pre-test probability of40%	pre-test probability of50%	pre-test probability of60%	
**True positives** (patients with an increase in cardiac output)	104 studies 2343 patients	cohort & case-control type studies	not serious	Not serious	not serious	serious[b]	none	308 (296 to 320)	385 (370 to 400)	462 (444 to 480)	⊕⊕⊕◯ Moderate
**False negatives** (patients incorrectly classified as not having an increase in cardiac output)								92 (80 to 104)	115 (100 to 130)	138 (120 to 156)	
**True negatives** (patients without an increase in cardiac output)	103 studies 2011 patients	cohort & case-control type studies	not serious	Not serious	not serious	serious[d]	none	474 (456 to 492)	395 (380 to 410)	316 (304 to 328)	⊕⊕⊕◯ Moderate
**False positives** (patients incorrectly classified as having an increase in cardiac output)								126 (108 to 144)	105 (90 to 120)	84 (72 to 96)	

Table S12. GRADE assessment for pulse pressure variation.

Question: Should Stroke volume variations be used to diagnose an increase in cardiac output in critical care patients after a fluid challenge?

Sensitivity	0.77 (95% CI: 0.73 to 0.80)
Specificity	0.78 (95% CI: 0.74 to 0.82)

Prevalence	40%	50%	60%

Outcome	№ of studies (№ of patients)	Study design	Factors that may decrease certainty of evidence					Effect per 1,000 patients tested			Test accuracy CoE
			Risk of bias	Indirectness	Inconsistency	Imprecision	Publication bias	pre-test probability of40%	pre-test probability of50%	pre-test probability of60%	
**True positives** (patients with an increase in cardiac output)	69 studies 1855 patients	cross-sectional (cohort type accuracy study)	not serious	not serious	not serious	not serious	none	308 (292 to 320)	385 (365 to 400)	462 (438 to 480)	⨁⨁⨁⨁ High
**False negatives** (patients incorrectly classified as not having an increase in cardiac output)								92 (80 to 108)	115 (100 to 135)	138 (120 to 162)	
**True negatives** (patients without an increase in cardiac output)	69 studies 1627 patients	cross-sectional (cohort type accuracy study)	not serious	not serious	not serious	not serious	none	468 (444 to 492)	390 (370 to 410)	312 (296 to 328)	⨁⨁⨁⨁ High
**False positives** (patients incorrectly classified as having an increase in cardiac output)								132 (108 to 156)	110 (90 to 130)	88 (72 to 104)	

Table S13. GRADE assessment for stroke volume variation.

Question: Should Passive leg raising be used to diagnose an increase in cardiac output in critical care patients after a fluid challenge?

Sensitivity	0.77 (95% CI: 0.73 to 0.80)
Specificity	0.80 (95% CI: 0.75 to 0.83)

Prevalence	40%	50%	60%

Outcome	№ of studies (№ of patients)	Study design	Factors that may decrease certainty of evidence					Effect per 1,000 patients tested			Test accuracy CoE
			Risk of bias	Indirectness	Inconsistency	Imprecision	Publication bias	pre-test probability of40%	pre-test probability of50%	pre-test probability of60%	
**True positives** (patients with an increase in cardiac output)	86 studies 2115 patients	cohort & case-control type studies	not serious	not serious	not serious	not serious	none	308 (292 to 320)	385 (365 to 400)	462 (438 to 480)	⊕⊕⊕⊕ High
**False negatives** (patients incorrectly classified as not having an increase in cardiac output)								92 (80 to 108)	115 (100 to 135)	138 (120 to 162)	
**True negatives** (patients without an increase in cardiac output)	83 studies 1998 patients	cohort & case-control type studies	not serious	not serious	not serious	not serious	none	0 (0 to 0)	0 (0 to 0)	0 (0 to 0)	⊕⊕⊕⊕ High
**False positives** (patients incorrectly classified as having an increase in cardiac output)								600 (600 to 600)	500 (500 to 500)	400 (400 to 400)	

Table S14. GRADE assessment for passive leg raising.

Question: Should end-expiratory occlusion test be used to diagnose increase in cardiac output in critical care patients after a fluid challenge?

Sensitivity	0.76 (95% CI: 0.70 to 0.81)
Specificity	0.77 (95% CI: 0.71 to 0.83)

Prevalence	40%	50%	60%

Outcome	№ of studies (№ of patients)	Study design	Factors that may decrease certainty of evidence					Effect per 1,000 patients tested			Test accuracy CoE
			Risk of bias	Indirectness	Inconsistency	Imprecision	Publication bias	pre-test probability of40%	pre-test probability of50%	pre-test probability of60%	
**True positives** (patients with increase in cardiac output)	36 studies 802 patients	cross-sectional (cohort type accuracy study)	not serious	not serious	not serious	not serious	none	304 (280 to 324)	380 (350 to 405)	456 (420 to 486)	⊕⊕⊕⊕ High
**False negatives** (patients incorrectly classified as not having increase in cardiac output)								96 (76 to 120)	120 (95 to 150)	144 (114 to 180)	
**True negatives** (patients without increase in cardiac output)	36 studies 836 patients	cross-sectional (cohort type accuracy study)	not serious	not serious	not serious	not serious	none	462 (426 to 498)	385 (355 to 415)	308 (284 to 332)	⊕⊕⊕⊕ High
**False positives** (patients incorrectly classified as having increase in cardiac output)								138 (102 to 174)	115 (85 to 145)	92 (68 to 116)	

Table S15. GRADE assessment for End-expiratory occlusion test.

Question: Should mini-fluid challenge be used to diagnose an increase in cardiac output in critical care patients after fluid challenge?

Sensitivity	0.85 (95% CI: 0.79 to 0.90)
Specificity	0.84 (95% CI: 0.78 to 0.88)

Prevalence	40%	50%	60%

Outcome	№ of studies (№ of patients)	Study design	Factors that may decrease certainty of evidence					Effect per 1,000 patients tested			Test accuracy CoE
			Risk of bias	Indirectness	Inconsistency	Imprecision	Publication bias	pre-test probability of40%	pre-test probability of50%	pre-test probability of60%	
**True positives** (patients with an increase in cardiac output)	16 studies 418 patients	cross-sectional (cohort type accuracy study)	not serious	not serious	not serious	not serious	none	340 (316 to 360)	425 (395 to 450)	510 (474 to 540)	⨁⨁⨁⨁ High
**False negatives** (patients incorrectly classified as not having an increase in cardiac output)								60 (40 to 84)	75 (50 to 105)	90 (60 to 126)	
**True negatives** (patients without an increase in cardiac output)	16 studies 457 patients	cross-sectional (cohort type accuracy study)	not serious	not serious	not serious	not serious	none	504 (468 to 528)	420 (390 to 440)	336 (312 to 352)	⨁⨁⨁⨁ High
**False positives** (patients incorrectly classified as having an increase in cardiac output)								96 (72 to 132)	80 (60 to 110)	64 (48 to 88)	

Table S16. GRADE assessment for mini-fluid challenge.

Question: Should tidal volume challenge be used to diagnose an increase in cardiac output in critical care patients after a fluid challenge?

Sensitivity	0.86 (95% CI: 0.81 to 0.91)
Specificity	0.80 (95% CI: 0.74 to 0.85)

Prevalence	40%	50%	60%

Outcome	№ of studies (№ of patients)	Study design	Factors that may decrease certainty of evidence					Effect per 1,000 patients tested			Test accuracy CoE
			Risk of bias	Indirectness	Inconsistency	Imprecision	Publication bias	pre-test probability of40%	pre-test probability of50%	pre-test probability of60%	
**True positives** (patients with an increase in cardiac output)	25 studies 518 patients	cohort & case-control type studies	not serious	not serious	not serious	not serious	none	344 (324 to 364)	430 (405 to 455)	516 (486 to 546)	⨁⨁⨁⨁ High
**False negatives** (patients incorrectly classified as not having an increase in cardiac output)								56 (36 to 76)	70 (45 to 95)	84 (54 to 114)	
**True negatives** (patients without an increase in cardiac output)	25 studies 483 patients	cohort & case-control type studies	not serious	not serious	not serious	not serious	none	480 (444 to 510)	400 (370 to 425)	320 (296 to 340)	⨁⨁⨁⨁ High
**False positives** (patients incorrectly classified as having an increase in cardiac output)								120 (90 to 156)	100 (75 to 130)	80 (60 to 104)	

Table S17. GRADE assessment for tidal-volume challenge.

Risk of bias within studies (QUADAS-2)	Predictor	Number of studies	Adjusted diagnostic odds ratios (95% CI)	P value by subgroup analysis	I^2 (%)
Low<br>High<br>Unclear	PPV	87<br>3<br>13	16.6 (12.5 – 22.0)<br>5.6 (1.6 – 19.1)<br>26.8 (12.1 – 59.4)	0.11	51.5
Low<br>High<br>Unclear	SVV	60<br>1<br>8	13.8 (10.1 – 18.8)<br>6.0(0.87-41.0)<br>26.5 (10.2 – 68.4)	0.29	51.4
Low<br>Unclear	PLR	79<br>4	22.9(17.0-30.9)<br>18.6(5-0-69.1)	0.76	55.4
Low<br>High	EOOT	34<br>4	18.7 (10.4-31.7)<br>17.7(3.49-84.5)	0.94	66.6
Low<br>High	MFC	14<br>2	38.9 (21.1 – 71.9)<br>32.3 (8.0 – 129.9)	0.8	34.3

Table S18. Sensitivity analysis of predictor of fluid responsiveness based on the methodological quality of the included studies. EEOT, end-expiratory occlusion test; I^2= inconsistency; PLR, passive leg raising; PPV, pulse pressure variation; MFC, mini-fluid challenge. QUADAS-2, quality assessment of diagnostic accuracy studies; SVV, strove volume variation. Values are expressed as pooled data (95% confidence interval)

Risk of bias within studies	Predictor	Number of studies	Adjusted diagnostic odds ratios (95% CI)	P value by test for subgroup	I^2 (%)	R^2 (%)
Measurement method to assess excitation	PPV					
method:		13	35.9(15.9-80.8)	0.06	50.60	10.37
PAC		2	17.7(3.3-94.6)			
PAC y TPTD(PiCCO)		12	19.7(9.4-41.3)			
Esophageal Doppler		1	40.0(1.8-890.4)			
TTE		10	38.5(16.6-89.3)			
TEE		1	15.11(1.24-184.75)			
PCA(LiCCO)		1	2173.0(28.8-164079.32)			
TPTD(LiCCO)		4	18.2(5.4-61.5)			
NC-PCA(Mostcare)		3	9.1(2.5-33.7)			
C-PCA(PiCCO)		32	10.3(6.6-15.9)			
TPTD(PiCCO)		2	4.3(0.9-19.0)			
NC-PCA(Pulsioflex/ProAQT)		14	18.5(9.2-37.0)			
NC-PCA(Vigileo)		6	16.3(5.6-47.4)			
CAP (Vigilance)		2	17.4(2.0-152.4)			
NC-PCA(ModelgFlow)						
Clinical setting	PPV					
Septic		39	19.32(12.4-30.1)	0.98	52.8	0.0
Surgical		22	13.61(8.1-23.0)			
Cardiovascular		26	16.16(9.5-27.6)			
Neurosurgical		5	16.31(8.3-32.1)			
Mix		1	12.5-22484.1)			
Number of patients included in each study	PPV	103	2.87(2.1-3.9)	0.15	53.5	9.4
Clinical setting	SVV					
Septic		15	17.7(10.9-28.8)	0.77	54.2	0.0
Surgical		21	8.12(4.9-13.3)			

Cardiovascular<br>Neurosurgical<br>Mix		21<br>10<br>2	21.0(12.4-35.7)<br>13.7(6.9-27.4)<br>19.71(3.5-109.4)			
Number of patients included in each study	SVV	69	2.75 +1.01(1.00-1.02)	0.18	58.2	3.16
Clinical Setting<br>Cardiovascular<br>Surgical<br>Septic<br>Mix	PLR	7<br>14<br>51<br>11	18.8(6.6-53.4)<br>23.8(11.3-50.0)<br>27.4(18.6-40.5)<br>12.8(6.0-27.2)	0.35	55.6	0.0
Number of patients included in each study	PLR	83	DOR=3.8(2.9-4.8)	0.44	64.6	1.55
Number of patients included in each study	EEOT	36	DOR = 6.75 – 0.99 (0.98-1.00)	0.04	62.2	14.2

Table S19. Sensitivity analysis of predictor of fluid responsiveness based on the method to measure cardiac output, clinical setting evaluated, hemodynamic variable to determine PLR/EEOT-response, device used to measure PLR response and the number of patients included in each study. I^2, inconsistency, R^2, amounts of heterogeneity; C-TD, continuous thermodilution; C-PCA, calibrated pulse contour analysis; CO, cardiac output; CI, cardiac index; EEOT, end-expiratory occlusion test; NC-PCA, non-calibrated pulse contour analysis; PAC, pulmonary artery catheter; PLR, passive leg raising; PPV, pulse pressure variation; SV, stroke volume; SVI, stroke volume index; SVV, stroke volume variation; TEE, transesophageal echocardiography; TPTD, transpulmonary thermodilution; TTE, transthoracic echocardiography; VTI, velocity time integral.

References

1. Michard F, Chemla D, Richard C, et al.: Clinical use of respiratory changes in arterial pulse pressure to monitor the hemodynamic effects of PEEP. *Am J Respir Crit Care Med* 1999; 159:935–939
2. Berkenstadt H, Margalit N, Hadani M, et al.: Stroke volume variation as a predictor of fluid responsiveness in patients undergoing brain surgery. *Anesth Analg* 2001; 92:984–989
3. Kramer A, Zygun D, Hawes H, et al.: Pulse pressure variation predicts fluid responsiveness following coronary artery bypass surgery. *Chest* 2004; 126:1563–1568
4. de Backer D, Heenen S, Piagnerelli M, et al.: Pulse pressure variations to predict fluid responsiveness: influence of tidal volume. *Intensive Care Med* 2005; 31:517–23
5. Feissel M, Badie J, Merlani PG, et al.: Pre-ejection period variations predict the fluid responsiveness of septic ventilated patients. *Crit Care Med* 2005; 33:2534–2539
6. Hofer CK, Müller SM, Furrer L, et al.: Stroke volume and pulse pressure variation for prediction of fluid responsiveness in patients undergoing off-pump coronary artery bypass grafting. *Chest* 2005; 128:848–854
7. Lafanechère A, Pène F, Goulenok C, et al.: Changes in aortic blood flow induced by passive leg raising predict fluid responsiveness in critically ill patients. *Crit Care* 2006; 10:1–8
8. Monnet X, Rienzo M, Osman D, et al.: Passive leg raising predicts fluid responsiveness in the critically ill. *Crit Care Med* 2006; 34:1402–1407
9. Charron C, Fessenmeyer C, Cosson C, et al.: The influence of tidal volume on the dynamic variables of fluid responsiveness in critically ill patients. *Anesth Analg* 2006; 102:1511–1517
10. Cannesson M, Attof Y, Rosamel P, et al.: Respiratory variations in pulse oximetry plethysmographic waveform amplitude to predict fluid responsiveness in the operating room. *Anesthesiology* 2007; 106:1105–1111
11. Feissel M, Teboul JL, Merlani P, et al.: Plethysmographic dynamic indices predict fluid responsiveness in septic ventilated patients. *Intensive Care Med* 2007; 33:993–999
12. Wyffels P a. H, Durnez P-J, Helderweirt J, et al.: Ventilation-Induced Plethysmographic Variations Predict Fluid Responsiveness in Ventilated Postoperative Cardiac Surgery Patients. *Anesth Analg* 2007; 105:448–452
13. Maizel J, Airapetian N, Lorne E, et al.: Diagnosis of central hypovolemia by using passive leg raising. *Intensive Care Med* 2007; 33:1133–1138
14. Lamia B, Ochagavia A, Monnet X, et al.: Echocardiographic prediction of volume responsiveness in critically ill patients with spontaneously breathing activity. *Intensive Care Med* 2007; 33:1125–1132
15. Auler JOJ, Galas F, Hajjar L, et al.: Online monitoring of pulse pressure variation to guide fluid therapy after cardiac surgery. *Anesth Analg* 2008; 106:1201–1206
16. Cannesson M, Desebbe O, Rosamel P, et al.: Pleth variability index to monitor the respiratory variations in the pulse oximeter plethysmographic waveform

amplitude and predict fluid responsiveness in the operating theatre. *Br J Anaesth* 2008; 101:200–206
17. Huang C-C, Fu J-Y, Hu H-C, et al.: Prediction of fluid responsiveness in acute respiratory distress syndrome patients ventilated with low tidal volume and high positive end-expiratory pressure. *Crit Care Med* 2008; 36:2810–2816
18. Derichard A, Robin E, Tavernier B, et al.: Automated pulse pressure and stroke volume variations from radial artery: evaluation during major abdominal surgery. *Br J Anaesth* 2009; 103:678–684
19. de Waal EEC, Rex S, Kruitwagen CLJJ, et al.: Dynamic preload indicators fail to predict fluid responsiveness in open-chest conditions*. *Crit Care Med* 2009; 37:510–515
20. Cannesson M, Musard H, Desebbe O, et al.: The ability of stroke volume variations obtained with vigileo/flotrac system to monitor fluid responsiveness in mechanically ventilated patients. *Anesth Analg* 2009; 108:513–517
21. Monge García MI, Gil Cano A, Díaz Monrové JC: Brachial artery peak velocity variation to predict fluid responsiveness in mechanically ventilated patients. *Crit Care* 2009; 13:1–9
22. Vistisen ST, Struijk JJ, Larsson A: Automated pre-ejection period variation indexed to tidal volume predicts fluid responsiveness after cardiac surgery. *Acta Anaesthesiol Scand* 2009; 53:534–542
23. Vallée F, Richard JCM, Mari A, et al.: Pulse pressure variations adjusted by alveolar driving pressure to assess fluid responsiveness. *Intensive Care Med* 2009; 35:1004–1010
24. Lahner D, Kabon B, Marschalek C, et al.: Evaluation of stroke volume variation obtained by arterial pulse contour analysis to predict fluid responsiveness intraoperatively. *Br J Anaesth* 2009; 103:346–351
25. Biais M, Vidil L, Sarrabay P, et al.: Changes in stroke volume induced by passive leg raising in spontaneously breathing patients: comparison between echocardiography and Vigileo/FloTrac device. *Crit Care* 2009; 13:R195
26. Thiel SW, Kollef MH, Isakow W: Non-invasive stroke volume measurement and passive leg raising predict volume responsiveness in medical ICU patients: an observational cohort study. *Crit Care* 2009; 13:R111
27. Monnet X, Osman D, Ridel C, et al.: Predicting volume responsiveness by using the end-expiratory occlusion in mechanically ventilated intensive care unit patients. *Crit Care Med* 2009; 37:951–6
28. Muller L, Louart G, Bousquet P-JJ, et al.: The influence of the airway driving pressure on pulsed pressure variation as a predictor of fluid responsiveness. *Intensive Care Med* 2010; 36:496–503
29. Biais M, Bernard O, Ha JC, et al.: Abilities of pulse pressure variations and stroke volume variations to predict fluid responsiveness in prone position during scoliosis surgery. *Br J Anaesth* 2010; 104:407–413
30. Zimmermann M, Feibicke T, Keyl C, et al.: Accuracy of stroke volume variation compared with pleth variability index to predict fluid responsiveness in mechanically ventilated patients undergoing major surgery. *Eur J Anaesthesiol* 2010; 27:555–561

31. Préau S, Saulnier F, Dewavrin F, et al.: Passive leg raising is predictive of fluid responsiveness in spontaneously breathing patients with severe sepsis or acute pancreatitis. *Crit Care Med* 2010; 38:819–825
32. Lakhal K, Ehrmann S, Benzekri-Lefèvre D, et al.: Respiratory pulse pressure variation fails to predict fluid responsiveness in acute respiratory distress syndrome. *Crit Care* 2011; 15:R85
33. Geerts BF, Maas J, de Wilde RBPP, et al.: Arm occlusion pressure is a useful predictor of an increase in cardiac output after fluid loading following cardiac surgery. *Eur J Anaesthesiol* 2011; 28:802–806
34. Desgranges F-P, Desebbe O, Ghazouani A, et al.: Influence of the site of measurement on the ability of plethysmographic variability index to predict fluid responsiveness. *Br J Anaesth* 2011; 107:329–335
35. Machare-Delgado E, Decaro M, Marik PE: Inferior vena cava variation compared to pulse contour analysis as predictors of fluid responsiveness: a prospective cohort study. *J Intensive Care Med* 2011; 26:116–124
36. Huang L, Zhang WX, Cai WX, et al.: Passive leg raising predicts volume responsiveness in patients with severe sepsis and septic shock. *Chinese Critical Care Medicine* 2011; 23:154–157
37. Broch O, Bein B, Gruenewald M, et al.: Accuracy of the pleth variability index to predict fluid responsiveness depends on the perfusion index. *Acta Anaesthesiol Scand* 2011; 55:686–693
38. Geerts B, de Wilde R, Aarts L, et al.: Pulse contour analysis to assess hemodynamic response to passive leg raising. *J Cardiothorac Vasc Anesth* 2011; 25:48–52
39. Loupec T, Nanadoumgar H, Frasca D, et al.: Pleth variability index predicts fluid responsiveness in critically ill patients. *Crit Care Med* 2011; 39:294–299
40. Muller L, Toumi M, Bousquet P-J, et al.: An Increase in Aortic Blood Flow after an Infusion of 100 ml Colloid over 1 Minute Can Predict Fluid Responsiveness The Mini-fluid Challenge Study. *Anesthesiology* 2011; 115:541–7
41. Fellahi J-L, Fischer M-O, Dalbera A, et al.: Can endotracheal bioimpedance cardiography assess hemodynamic response to passive leg raising following cardiac surgery? *Ann Intensive Care* 2012; 2:1–12
42. Fellahi JL, Fischer MO, Rebet O, et al.: A comparison of endotracheal bioimpedance cardiography and transpulmonary thermodilution in cardiac surgery patients. *J Cardiothorac Vasc Anesth* 2012; 26:217–222
43. Khwannimit B, Bhurayanontachai R: Prediction of fluid responsiveness in septic shock patients: comparing stroke volume variation by FloTrac/Vigileo and automated pulse pressure variation. *Eur J Anaesthesiol* 2012; 29:64–69
44. Monnet X, Bleibtreu A, Ferré A, et al.: Passive leg-raising and end-expiratory occlusion tests perform better than pulse pressure variation in patients with low respiratory system compliance. *Crit Care Med* 2012; 40:152–7
45. Monnet X, Dres M, Ferré A, et al.: Prediction of fluid responsiveness by a continuous non-invasive assessment of arterial pressure in critically ill patients: Comparison with four other dynamic indices. *Br J Anaesth* 2012; 109:330–338

46. Cecconi M, Monti G, Hamilton MA, et al.: Efficacy of functional hemodynamic parameters in predicting fluid responsiveness with pulse power analysis in surgical patients. *Minerva Anestesiol* 2012; 78:527–533
47. Yazigi A, Khoury E, Hlais S, et al.: Pulse pressure variation predicts fluid responsiveness in elderly patients after coronary artery bypass graft surgery. *J Cardiothorac Vasc Anesth* 2012; 26:387–390
48. Oliveira-Costa CDA, Friedman G, Vieira SRR, et al.: Pulse pressure variation and prediction of fluid responsiveness in patients ventilated with low tidal volumes. *Clinics (Sao Paulo)* 2012; 67:773–778
49. Biais M, Cottenceau V, Stecken L, et al.: Evaluation of stroke volume variations obtained with the pressure recording analytic method. *Crit Care Med* 2012; 40:1186–1191
50. Fu Q, Mi WD, Zhang H: Stroke volume variation and pleth variability index to predict fluid responsiveness during resection of primary retroperitoneal tumors in Hans Chinese. *Biosci Trends* 2012; 6:38–43
51. Li J, Ji FH, Yang JP: Evaluation of stroke volume variation obtained by the flotrac^{TM}/Vigileo^{TM} system to guide preoperative fluid therapy in patients undergoing brain surgery. *J Int Med Res* 2012; 40:1175–1181
52. Dong Z-Z, Fang Q, Zheng X, et al.: Passive leg raising as an indicator of fluid responsiveness in patients with severe sepsis. *World J Emerg Med* 2012; 3:191–196
53. Monge García MI, Cano AG, Romero MG, et al.: Non-invasive assessment of fluid responsiveness by changes in partial end-tidal CO2 pressure during a passive leg-raising maneuver. *Ann Intensive Care* 2012; 2:9
54. Broch O, Renner J, Gruenewald M, et al.: Variation of left ventricular outflow tract velocity and global end-diastolic volume index reliably predict fluid responsiveness in cardiac surgery patients. *J Crit Care* 2012; 27:325.e7-325.e13
55. Monnet X, Guérin L, Jozwiak M, et al.: Pleth variability index is a weak predictor of fluid responsiveness in patients receiving norepinephrine. *Br J Anaesth* 2013; 110:207–213
56. Monnet X, Bataille AA, Magalhaes E, et al.: End-tidal carbon dioxide is better than arterial pressure for predicting volume responsiveness by the passive leg raising test. *Intensive Care Med* 2013; 39:93–100
57. Davies SJ, Minhas S, Wilson RJT, et al.: Comparison of stroke volume and fluid responsiveness measurements in commonly used technologies for goal-directed therapy. *J Clin Anesth* 2013; 25:466–474
58. Nordström J, Hällsjö-Sander C, Shore R, et al.: Stroke volume optimization in elective bowel surgery: A comparison between pulse power wave analysis (LiDCOrapid) and oesophageal Doppler (CardioQ). *Br J Anaesth* 2013; 110:374–380
59. Fischer MO, Coucoravas J, Truong J, et al.: Assessment of changes in cardiac index and fluid responsiveness: A comparison of Nexfin and transpulmonary thermodilution. *Acta Anaesthesiol Scand* 2013; 57:704–712
60. Ishihara H, Hashiba E, Okawa H, et al.: Neither dynamic, static, nor volumetric variables can accurately predict fluid responsiveness early after abdominothoracic esophagectomy. *Perioperative Medicine* 2013; 2:1–8

61. Drvar Z, Tonković D, Pavlek M, et al.: Stroke volume variation and pulse pressure variation as predictors of fl uid responsiveness in patients with sepsis. *Neurologia Croatica* 2013; 62:1–4
62. Freitas FGR, Bafi AT, Nascente APM, et al.: Predictive value of pulse pressure variation for fluid responsiveness in septic patients using lung-protective ventilation strategies. *Br J Anaesth* 2013; 110:402–408
63. Trepte CJC, Eichhorn V, Haas SA, et al.: Comparison of an automated respiratory systolic variation test with dynamic preload indicators to predict fluid responsiveness after major surgery. *Br J Anaesth* 2013; 111:736–742
64. Yang S-Y, Shim J-K, Song Y, et al.: Validation of pulse pressure variation and corrected flow time as predictors of fluid responsiveness in patients in the prone position. *Br J Anaesth* 2013; 110:713–720
65. Kim SY, Song Y, Shim JK, et al.: Effect of pulse pressure on the predictability of stroke volume variation for fluid responsiveness in patients with coronary disease. *J Crit Care* 2013; 28:318.e1–7
66. Saugel B, Kirsche S v, Hapfelmeier A, et al.: Prediction of fluid responsiveness in patients admitted to the medical intensive care unit. *J Crit Care* 2013; 28:537.e1–9
67. Kupersztych-Hagege E, Teboul J-L, Artigas A, et al.: Bioreactance is not reliable for estimating cardiac output and the effects of passive leg raising in critically ill patients. *Br J Anaesth* 2013; 111:961–966
68. Silva S, Jozwiak M, Teboul J-L, et al.: End-Expiratory Occlusion Test Predicts Preload Responsiveness Independently of Positive End-Expiratory Pressure During Acute Respiratory Distress Syndrome. *Crit Care Med* 2013; 41:1692–1701
69. Pei S, Yuan W, Mai H, et al.: Efficacy of dynamic indices in predicting fluid responsiveness in patients with obstructive jaundice. *Physiol Meas* 2014; 35:369–382
70. Høiseth LØ, Hoff IE, Hagen OA, et al.: Dynamic variables and fluid responsiveness in patients for aortic stenosis surgery. *Acta Anaesthesiol Scand* 2014; 58:826–834
71. Guarracino F, Ferro B, Forfori F, et al.: Jugular vein distensibility predicts fluid responsiveness in septic patients. *Crit Care* 2014; 18:1–8
72. Song Y, Kwak YL, Song JW, et al.: Respirophasic carotid artery peak velocity variation as a predictor of fluid responsiveness in mechanically ventilated patients with coronary artery disease. *Br J Anaesth* 2014; 113:61–66
73. Shim J-K, Song J-W, Song Y, et al.: Pulse pressure variation is not a valid predictor of fluid responsiveness in patients with elevated left ventricular filling pressure. *J Crit Care* 2014; 29:987–91
74. Fischer M-O, Pellissier A, Saplacan V, et al.: Cephalic versus digital plethysmographic variability index measurement: a comparative pilot study in cardiac surgery patients. *J Cardiothorac Vasc Anesth* 2014; 28:1510–1515
75. Kurtz P, Helbok R, Ko S-B, et al.: Fluid responsiveness and brain tissue oxygen augmentation after subarachnoid hemorrhage. *Neurocrit Care* 2014; 20:247–254
76. Guinot P-G, de Broca B, Bernard E, et al.: Respiratory stroke volume variation assessed by oesophageal Doppler monitoring predicts fluid responsiveness during laparoscopy. *Br J Anaesth* 2014; 112:660–664

77. Kang W-S, Kim S-H, Kim SY, et al.: The influence of positive end-expiratory pressure on stroke volume variation in patients undergoing cardiac surgery: An observational study. *Journal of Thoracic and Cardiovascular Surgery* 2014; 148:3139–3145
78. Guinot P-GG, Godart J, de Broca B, et al.: End-expiratory occlusion manoeuvre does not accurately predict fluid responsiveness in the operating theatre. *Br J Anaesth* 2014; 112:1050–1054
79. Wu Y, Zhou S, Zhou Z, et al.: A 10-second fluid challenge guided by transthoracic echocardiography can predict fluid responsiveness. *Crit Care* 2014; 18:1–8
80. Messina A, Colombo D, Cammarota G, et al.: Patient-ventilator asynchrony affects pulse pressure variation prediction of fluid responsiveness. *J Crit Care* 2015; 30:1067–1071
81. Zhao F, Wang P, Pei S, et al.: Automated stroke volume and pulse pressure variations predict fluid responsiveness in mechanically ventilated patients with obstructive jaundice. *Int J Clin Exp Med* 2015; 8:20751–20759
82. Ibarra-Estrada M, López-Pulgarín JA, Mijangos-Méndez JC, et al.: Respiratory variation in carotid peak systolic velocity predicts volume responsiveness in mechanically ventilated patients with septic shock: a prospective cohort study. *Crit Ultrasound J* 2015; 7:1–9
83. Angappan S, Parida S, Vasudevan A, et al.: The comparison of stroke volume variation with central venous pressure in predicting fluid responsiveness in septic patients with acute circulatory failure. *Indian Journal of Critical Care Medicine* 2015; 19:394–400
84. Airapetian N, Maizel J, Alyamani O, et al.: Does inferior vena cava respiratory variability predict fluid responsiveness in spontaneously breathing patients? *Crit Care* 2015; 19:400
85. Guinot PG, Bernard E, Defrancq F, et al.: Mini-fluid challenge predicts fluid responsiveness during spontaneous breathing under spinal anaesthesia: An observational study. *Eur J Anaesthesiol* 2015; 32:645–649
86. Mallat J, Meddour M, Durville E, et al.: Decrease in pulse pressure and stroke volume variations after mini-fluid challenge accurately predicts fluid responsiveness. *Br J Anaesth* 2015; 115:449–456
87. Theerawit P, Morasert T, Sutherasan Y: Inferior vena cava diameter variation compared with pulse pressure variation as predictors of fluid responsiveness in patients with sepsis. *J Crit Care* 2016; 36:246–251
88. Lee SH, Chun Y-MM, Oh YJ, et al.: Prediction of fluid responsiveness in the beach chair position using dynamic preload indices. *J Clin Monit Comput* 2016; 30:995–1002
89. Cherpanath TGV, Geerts BF, Maas JJ, et al.: Ventilator-induced central venous pressure variation can predict fluid responsiveness in post-operative cardiac surgery patients. *Acta Anaesthesiol Scand* 2016; 60:1395–1403
90. Zhang X, Feng J, Zhu P, et al.: Ultrasonographic measurements of the inferior vena cava variation as a predictor of fluid responsiveness in patients undergoing anesthesia for surgery. *J Surg Res* 2016; 204:118–122

91. Fischer M-O, Rebet O, Guinot P-G, et al.: Assessment of changes in cardiac index with calibrated pulse contour analysis in cardiac surgery: A prospective observational study. *Anaesth Crit Care Pain Med* 2016; 35:261–267
92. Kim N, Shim J-K, Choi HG, et al.: Comparison of positive end-expiratory pressure-induced increase in central venous pressure and passive leg raising to predict fluid responsiveness in patients with atrial fibrillation. *Br J Anaesth* 2016; 116:350–356
93. de Oliveira OH, Freitas FGR de, Ladeira RT, et al.: Comparison between respiratory changes in the inferior vena cava diameter and pulse pressure variation to predict fluid responsiveness in postoperative patients. *J Crit Care* 2016; 34:46–49
94. Sobczyk D, Nycz K, Andruszkiewicz P, et al.: Ultrasonographic caval indices do not significantly contribute to predicting fluid responsiveness immediately after coronary artery bypass grafting when compared to passive leg raising. *Cardiovasc Ultrasound* 2016; 14:1–8
95. Liu Y, Wei L, Li G, et al.: Pulse Pressure Variation Adjusted by Respiratory Changes in Pleural Pressure, Rather Than by Tidal Volume, Reliably Predicts Fluid Responsiveness in Patients With Acute Respiratory Distress Syndrome. *Crit Care Med* 2016; 44:342–51
96. Vistisen ST: Using extra systoles to predict fluid responsiveness in cardiothoracic critical care patients. *J Clin Monit Comput* 2017; 31:693–699
97. Jacquet-Lagrèze M, Baudin F, David JS, et al.: End-tidal carbon dioxide variation after a 100- and a 500-ml fluid challenge to assess fluid responsiveness. *Ann Intensive Care* 2016; 6
98. Krige A, Bland M, Fanshawe T: Fluid responsiveness prediction using Vigileo FloTrac measured cardiac output changes during passive leg raise test. *J Intensive Care* 2016; 4
99. Myatra SN, Prabu NR, Divatia JV, et al.: The Changes in Pulse Pressure Variation or Stroke Volume Variation After a “Tidal Volume Challenge” Reliably Predict Fluid Responsiveness During Low Tidal Volume Ventilation. *Crit Care Med* 2017; 415–421
100. Yonis H, Bitker L, Aublanc M, et al.: Change in cardiac output during Trendelenburg maneuver is a reliable predictor of fluid responsiveness in patients with acute respiratory distress syndrome in the prone position under protective ventilation. *Crit Care* 2017; 21:1–12
101. Renner J, Gruenewald M, Hill M, et al.: Non-invasive assessment of fluid responsiveness using CNAP™ technology is interchangeable with invasive arterial measurements during major open abdominal surgery. *Br J Anaesth* 2017; 118:58–67
102. Min JJ, Lee J-H, Hong KY, et al.: Utility of stroke volume variation measured using non-invasive bioreactance as a predictor of fluid responsiveness in the prone position. *J Clin Monit Comput* 2017; 31:397–405
103. Biais M, Larghi M, Henriot J, et al.: End-Expiratory Occlusion Test Predicts Fluid Responsiveness in Patients With Protective Ventilation in the Operating Room. *Anesth Analg* 2017; 125:1889–1895

104. Biais M, de Courson H, Lanchon R, et al.: Mini-fluid Challenge of 100 ml of Crystalloid Predicts Fluid Responsiveness in the Operating Room. *Anesthesiology* 2017; 127:450–456
105. Messina A, Romano SM, Bonicolini E, et al.: Cardiac cycle efficiency and dicrotic pressure variations: New parameters for fluid therapy: An observational study. *Eur J Anaesthesiol* 2017; 34:755–763
106. Lu N, Xi X, Jiang L, et al.: Exploring the best predictors of fluid responsiveness in patients with septic shock. *Am J Emerg Med* 2017; 35:1258–1261
107. el Hadouti Y, Valencia L, Becerra A, et al.: Echocardiography and passive leg raising in the postoperative period: A prospective observational study. *Eur J Anaesthesiol* 2017; 34:748–754
108. Jozwiak M, Depret F, Teboul JL, et al.: Predicting Fluid Responsiveness in Critically Ill Patients by Using Combined End-Expiratory and End-Inspiratory Occlusions With Echocardiography. *Crit Care Med* 2017; 45:e1131–e1138
109. Renner J, Gruenewald M, Hill M, et al.: Non-invasive assessment of fluid responsiveness using CNAP^{TM} technology is interchangeable with invasive arterial measurements during major open abdominal surgery. *Br J Anaesth* 2017; 118:58–67
110. Xu J, Peng X, Pan C, et al.: Fluid responsiveness predicted by transcutaneous partial pressure of oxygen in patients with circulatory failure: a prospective study. *Ann Intensive Care* 2017; 7
111. Ma G guang, Hao G wei, Yang X mei, et al.: Internal jugular vein variability predicts fluid responsiveness in cardiac surgical patients with mechanical ventilation. *Ann Intensive Care* 2018; 8
112. Wu J, Wang Z, Wang T, et al.: Evaluation of the fluid responsiveness in patients with septic shock by ultrasound plus the passive leg raising test [Internet]. *Journal of Surgical Research* 2018; 224:207–214Available from: https://doi.org/10.1016/j.jss.2017.12.014
113. Si X, Cao D-Y, Chen J, et al.: Effect of Systolic Cardiac Function on Passive Leg Raising for Predicting Fluid Responsiveness: A Prospective Observational Study. *Chin Med J (Engl)* 2018; 131:253–261
114. Georges D, de Courson H, Lanchon R, et al.: End-expiratory occlusion maneuver to predict fluid responsiveness in the intensive care unit: An echocardiographic study. *Crit Care* 2018; 22:1–8
115. Joosten A, Boudart C, Vincent J-L, et al.: Ability of a New Smartphone Pulse Pressure Variation and Cardiac Output Application to Predict Fluid Responsiveness in Patients Undergoing Cardiac Surgery. *Anesth Analg* 2019; 128:1145–1151
116. Ali A, Abdullah T, Sabanci PA, et al.: Comparison of ability of pulse pressure variation to predict fluid responsiveness in prone and supine position: an observational study. *J Clin Monit Comput* 2019; 33:573–580
117. He F, Li X, Thapa S, et al.: Evaluation of volume responsiveness by pulse pressure variability and inferior vena cava dispensability index at different tidal volumes by mechanical ventilation. *Brazilian journal of medical and biological research* 2019; 52:e8827

118. Ali A, Dorman Y, Abdullah T, et al.: Ability of mini-fluid challenge to predict fluid responsiveness in obese patients undergoing surgery in the prone position. *Minerva Anestesiol* 2019; 85:981–988
119. Xu L-Y, Tu G-W, Cang J, et al.: End-expiratory occlusion test predicts fluid responsiveness in cardiac surgical patients in the operating theatre. *Ann Transl Med* 2019; 7
120. Trifi A, Abdellatif S, Daly F, et al.: Ultrasound stroke volume variation induced by passive leg raising and fluid responsiveness: An observational cohort study. *Med Intensiva* 2019; 43:10–17
121. Beurton A, Teboul J-LL, Girotto V, et al.: Intra-Abdominal Hypertension Is Responsible for False Negatives to the Passive Leg Raising Test. *Crit Care Med* 2019; 47:E639–E647
122. Dépret F, Jozwiak M, Teboul J-LJL, et al.: Esophageal Doppler Can Predict Fluid Responsiveness Through End-Expiratory and End-Inspiratory Occlusion Tests [Internet]. *Crit Care Med* 2019; 47:e96–e102Available from: https://www.embase.com/search/results?subaction=viewrecord&id=L625988583&from=export
123. Messina A, Montagnini C, Cammarota G, et al.: Tidal volume challenge to predict fluid responsiveness in the operating room: An observational study. *Eur J Anaesthesiol* 2019; 36:583–591
124. Fot E v., Izotova NN, Smetkin AA, et al.: Dynamic Tests to Predict Fluid Responsiveness After Off-Pump Coronary Artery Bypass Grafting. *J Cardiothorac Vasc Anesth* 2020; 34:926–931
125. Beurton A, Teboul JL, Gavelli F, et al.: The effects of passive leg raising may be detected by the plethysmographic oxygen saturation signal in critically ill patients. *Crit Care* 2019; 23
126. Jun JH, Chung RK, Baik HJ, et al.: The tidal volume challenge improves the reliability of dynamic preload indices during robot-assisted laparoscopic surgery in the Trendelenburg position with lung-protective ventilation. *BMC Anesthesiol* 2019; 19
127. Lee C-T, Lee T, Chiu C-T, et al.: Mini- fluid challenge test predicts stroke volume and arterial pressure fluid responsiveness during spine surgery in prone position. *Medicine* 2020; 99:e19031
128. Wang J, Zhou D, Gao Y, et al.: Effect of VTILVOT variation rate on the assessment of fluid responsiveness in septic shock patients. *Medicine* 2020; 99:e22702
129. Bapteste L, Carrillon R, Javelier S, et al.: Pulse Pressure Variations and Plethysmographic Variability Index Measured at Ear Are Able to Predict Fluid Responsiveness in the Sitting Position for Neurosurgery. *J Neurosurg Anesthesiol* 2020; 32:263–267
130. Bubenek-Turconi Ş-I, Hendy A, Băilă S, et al.: The value of a superior vena cava collapsibility index measured with a miniaturized transoesophageal monoplane continuous echocardiography probe to predict fluid responsiveness compared to stroke volume variations in open major vascular surgery: a prospe. *J Clin Monit Comput* 2020; 34:491–499

131. Hou J-Y, Zheng J-L, Ma G-G, et al.: Evaluation of radial artery pulse pressure effects on detection of stroke volume changes after volume loading maneuvers in cardiac surgical patients. *Ann Transl Med* 2020; 8
132. Weil G, Motamed C, Monnet X, et al.: End-Expiratory Occlusion Test to Predict Fluid Responsiveness Is Not Suitable for Laparotomic Surgery. *Anesth Analg* 2020; 130:151–158
133. Messina A, Montagnini C, Cammarota G, et al.: Assessment of fluid responsiveness in prone neurosurgical patients undergoing protective ventilation: Role of dynamic indices, tidal volume challenge, and end-expiratory occlusion test. *Anesth Analg* 2020; 752–761
134. Hamzaoui O, Shi R, Carelli S, et al.: Changes in pulse pressure variation to assess preload responsiveness in mechanically ventilated patients with spontaneous breathing activity: an observational study. *Br J Anaesth* 2021; 127:532–538
135. Giraud R, Vujovic B, Assouline B, et al.: Do ScvO2 variations induced by passive leg raising predict fluid responsiveness? A prospective study. *Physiol Rep* 2021; 9
136. Kaur K, Nakra M, Mangal V, et al.: Comparative evaluation of stroke volume variation and inferior vena cava distensibility index for prediction of fluid responsiveness in mechanically ventilated patients. *Ann Card Anaesth* 2021; 24:327–332
137. Bataille B, de Selle J, Moussot P-E, et al.: Machine learning methods to improve bedside fluid responsiveness prediction in severe sepsis or septic shock: an observational study. *Br J Anaesth* 2021; 126:826–834
138. Lizuka Y, Sanui M, Otsuka Y, et al.: Changes in peripheral perfusion index during intraoperative end-expiratory occlusion tests do not predict the response to fluid administration in patients undergoing lung protective ventilation. *J Anesth* 2021; 35:837–843
139. Messina A, Lionetti G, Foti L, et al.: Mini fluid chAllenge and End-expiratory occlusion test to assess flUid responsiVEness in the opeRating room (MANEUVER study): A multicentre cohort study. *Eur J Anaesthesiol* 2021; 38:422–431
140. Watanabe R, Suehiro K, Mukai A, et al.: Changes in stroke volume induced by lung recruitment maneuver can predict fluid responsiveness during intraoperative lung-protective ventilation in prone position. *BMC Anesthesiol* 2021; 21
141. Chen Y-H, Lai Y-J, Huang C-Y, et al.: Effects of positive end-expiratory pressure on the predictability of fluid responsiveness in acute respiratory distress syndrome patients. *Sci Rep* 2021; 11:10186
142. Taccheri T, Gavelli F, Teboul JL, et al.: Do changes in pulse pressure variation and inferior vena cava distensibility during passive leg raising and tidal volume challenge detect preload responsiveness in case of low tidal volume ventilation? *Crit Care* 2021; 25
143. Elsayed AI, Selim KAW, Zaghla HE, et al.: Comparison of changes in PPV using a tidal volume challenge with a passive leg raising test to predict fluid responsiveness in patients ventilated using low tidal volume. *Indian Journal of Critical Care Medicine* 2021; 25:685–689

144. Shi R, Moretto F, Prat D, et al.: Dynamic changes of pulse pressure but not of pulse pressure variation during passive leg raising predict preload responsiveness in critically ill patients with spontaneous breathing activity. *J Crit Care* 2022; 72
145. Shi R, Ayed S, Moretto F, et al.: Tidal volume challenge to predict preload responsiveness in patients with acute respiratory distress syndrome under prone position. *Crit Care* 2022; 26
146. Abdullah T, Ali A, Saka E, et al.: Ability of short-time low peep challenge to predict fluid responsiveness in mechanically ventilated patients in the intensive care. *J Clin Monit Comput* 2022; 36:1165–1172
147. Xu Y, Guo J, Wu Q, et al.: Efficacy of using tidal volume challenge to improve the reliability of pulse pressure variation reduced in low tidal volume ventilated critically ill patients with decreased respiratory system compliance. *BMC Anesthesiol* 2022; 22
148. Mallat J, Fischer MO, Granier M, et al.: Passive leg raising-induced changes in pulse pressure variation to assess fluid responsiveness in mechanically ventilated patients: a multicentre prospective observational study. *Br J Anaesth* 2022; 129:308–316
149. Salem YSM, Botros JM, Khalil M, et al.: Effects of tidal volume challenge on the reliability of plethysmography variability index in hepatobiliary and pancreatic surgeries: a prospective interventional study. *Preprint* 2022;
